# Silencing of transcription factor encoding gene *StTCP23* by small RNAs derived from the virulence modulating region of *potato spindle tuber viroid* is associated with symptom development in potato

**DOI:** 10.1371/journal.ppat.1008110

**Published:** 2019-12-02

**Authors:** Sarina Bao, Robert A. Owens, Qinghua Sun, Hui Song, Yanan Liu, Andrew Leigh Eamens, Hao Feng, Hongzhi Tian, Ming-Bo Wang, Ruofang Zhang

**Affiliations:** 1 School of Life Sciences, Inner Mongolia University, Hohhot, China; 2 Molecular Plant Pathology Laboratory, USDA/ARS, Beltsville, Maryland, United States of America; 3 Centre for Plant Science, School of Environmental and Life Sciences, Faculty of Science, University of Newcastle, Australia; 4 CSIRO Agriculture and Food, Canberra, Australia; Agriculture and Agri-Food Canada, CANADA

## Abstract

Viroids are small, non-protein-coding RNAs which can induce disease symptoms in a variety of plant species. Potato (*Solanum tuberosum* L.) is the natural host of *Potato spindle tuber viroid* (PSTVd) where infection results in stunting, distortion of leaves and tubers and yield loss. Replication of PSTVd is accompanied by the accumulation of viroid-derived small RNAs (sRNAs) proposed to play a central role in disease symptom development. Here we report that PSTVd sRNAs direct RNA silencing in potato against *StTCP23*, a member of the TCP (teosinte branched1/Cycloidea/Proliferating cell factor) transcription factor family genes that play an important role in plant growth and development as well as hormonal regulation, especially in responses to gibberellic acid (GA). The *StTCP23* transcript has 21-nucleotide sequence complementarity in its 3ʹ untranslated region with the virulence-modulating region (VMR) of PSTVd strain RG1, and was downregulated in PSTVd-infected potato plants. Analysis using 3ʹ RNA ligase-mediated rapid amplification of cDNA ends (3ʹ RLM RACE) confirmed cleavage of *StTCP23* transcript at the expected sites within the complementarity with VMR-derived sRNAs. Expression of these VMR sRNA sequences as artificial miRNAs (amiRNAs) in transgenic potato plants resulted in phenotypes reminiscent of PSTVd-RG1-infected plants. Furthermore, the severity of the phenotypes displayed was correlated with the level of amiRNA accumulation and the degree of amiRNA-directed down-regulation of *StTCP23*. In addition, virus-induced gene silencing (VIGS) of *StTCP23* in potato also resulted in PSTVd-like phenotypes. Consistent with the function of TCP family genes, amiRNA lines in which *StTCP23* expression was silenced showed a decrease in GA levels as well as alterations to the expression of GA biosynthesis and signaling genes previously implicated in tuber development. Application of GA to the amiRNA plants minimized the PSTVd-like phenotypes. Taken together, our results indicate that sRNAs derived from the VMR of PSTVd-RG1 direct silencing of *StTCP23* expression, thereby disrupting the signaling pathways regulating GA metabolism and leading to plant stunting and formation of small and spindle-shaped tubers.

## Author summary

*Potato spindle tuber viroid* (PSTVd) is a small RNA pathogen that causes severe pandemic diseases in potato. How this non-protein-coding RNA induces disease symptom development in potato is unknown, thereby hindering the development of effective control measures. Here we report the first evidence that PSTVd disease is caused by the silencing of *StTCP23*, a potato transcription factor encoding gene, by PSTVd-derived small-interfering RNA (siRNAs). Specifically, we demonstrate that 3ʹ untranslated region (UTR) region of *StTCP23* mRNA contains a 21-nt sequence that is complementary to the virulence-modulating region (VMR) of PSTVd. Furthermore, we show that *StTCP23* expression is repressed in PSTVd-infected potato, and this repression is accompanied by *StTCP23* transcript cleavage within the identified region of complementary. *In planta* expression of VMR sequences as 21-nt artificial microRNAs (amiRNAs) or infection of potato plants with a virus-induced gene silencing vector containing a portion the *StTCP23* coding sequence, results in reduced *StTCP23* transcript abundance and the expression of PSTVd-like disease symptoms. Consistent with the predicted functional role of *St*TCP23 in regulating the gibberellic acid (GA) biosynthesis and signaling pathways, GA levels were reduced both in PSTVd-infected and amiRNA-expressing plants. Our results provide compelling evidence that *St*TCP23 positively regulates potato sprouting and tuber development via a GA-related mechanism, and that the disease symptoms that develop upon PSTVd infection result from silencing of *StTCP23* by VMR-derived siRNAs.

## Introduction

Viroids are small, non-protein-coding RNA pathogens whose genomes range in size from 246 to 401 nucleotides. *Potato spindle tuber viroid* (PSTVd), the causal agent of the “spindle tuber” disease of potato (*Solanum tuberosum* L.), is a member of the viroid family *Pospiviroidae* [1, 2]. PSTVd replicates in the nucleus and moves systemically throughout the plant in the phloem, with its infection resulting in the development of a wide array of symptoms [3]. Stunting is a common phenotypic outcome of PSTVd infection, and in the case of potato, the tubers of infected plants are reduced in size with an elongated or spindle-shaped morphology. In addition, such tubers have prominent eyes evenly distributed over their entire surface. Tubers from infected plants sprout more slowly than those from healthy non-infected plants, and infected plantlets exhibit a variety of symptoms [4–6].

As a vegetatively propagated crop, the quality requirements of seed potatoes are extremely high. Propagation by tubers, cuttings, and micropropagation of plants is highly conducive for PSTVd transmission [7, 8], and once established, the infection is persistent and extremely difficult to eliminate by conventional methods such as stem tip stripping [9]. PSTVd is also transmitted by pollen and true potato seed during germplasm collection and hybridization-based breeding [10]. Despite the threat posed to the production of both seed and ware potatoes, the mechanism of disease symptom development upon PSTVd infection remains poorly understood.

RNA silencing provides a multi-layered defense system that in plants provides protection from invasion by exogenous RNA replicons, such as viruses and viroids [11]. RNA silencing is triggered by the conversion of long double-stranded RNAs (dsRNAs) into small RNAs (sRNAs) of approximately 21 to 24 nucleotides (nt) in length and the accumulation of such viroid-derived sRNAs (vd-sRNAs) has been extensively studied for several different viroid-host combinations [12, 13]. Their high degree of internal base pairing and RNA-RNA mode of replication make viroids a potent trigger of sRNA-directed RNA silencing with infected hosts often containing extremely high levels of vd-siRNA [11, 12, 14, 15]. The generated vd-sRNAs are bound by multiple plant argonaute proteins, thereby supporting the hypothesis that viroid infection triggers vd-sRNA-directed RNA silencing and that this process plays a role in disease symptom development [16].

PSTVd contains five structural domains, including the terminal left region, pathogenicity domain, central conserved region, variable region, and the terminal right region [16]. Within the pathogenicity domain, it has been shown that even a single nucleotide change within the so-called *virulence modulating region* (VMR) can result in dramatic differences in the severity of the disease symptoms displayed [17, 18]. In *Nicotiana* species, an artificial microRNA corresponding to the VMR of the PSTVd-RG1 strain directed RNA silencing of a *soluble inorganic pyrophosphatase* gene and the development of abnormal phenotypes [19]. A study by Adkar-Purushothama et al. showed that the sRNAs derived from the same region of the PSTVd-Intermediate strain down-regulated the expression of a *callose synthase* gene in tomato and altered the severity of the induced disease symptom [20]. A subsequent in silico study by the same authors indicated that siRNAs derived from the VMR may also potentially modulate the expression of a *serine threonine kinase receptor* gene to regulate disease resistance in tomato [21]. Furthermore, a study by Katsarou et al. has shown that PSTVd infection alters the expression of hormonal pathway-related genes in potato, including the *gibberellin 7-oxidase* (*GA7ox*) *and gibberellin-insensitive dwarf protein 1* (*GID1*) genes, although viroid-derived sRNAs were not found to be responsible [22].

Here we report the identification of a potato transcription factor that shows sequence homology to the VMR region of PSTVd. We examined the possible targeting of the identified transcription factor, *StTCP23*, for RNA silencing by sRNAs derived from the PSTVd genome, demonstrating that *StTCP23* expression was reduced by PSTVd infection, which correlated with the cleavage of *StTCP23* mRNA at the complementary region. We also show that transgenic potato plants expressing artificial microRNAs derived from the complementary VMR sequences displayed PSTVd-like phenotypes. In addition, silencing of *StTCP23* using a virus-induced gene silencing vector also resulted in phenotypes that were highly similar to those observed in PSTVd-infected plants. Our data strongly indicate that PSTVd-induced disease phenotypes in potato is due to the silencing of *StTCP23* by VMR-derived sRNA that alters the GA biosynthesis and signaling pathway.

## Results

**Fig 1:**
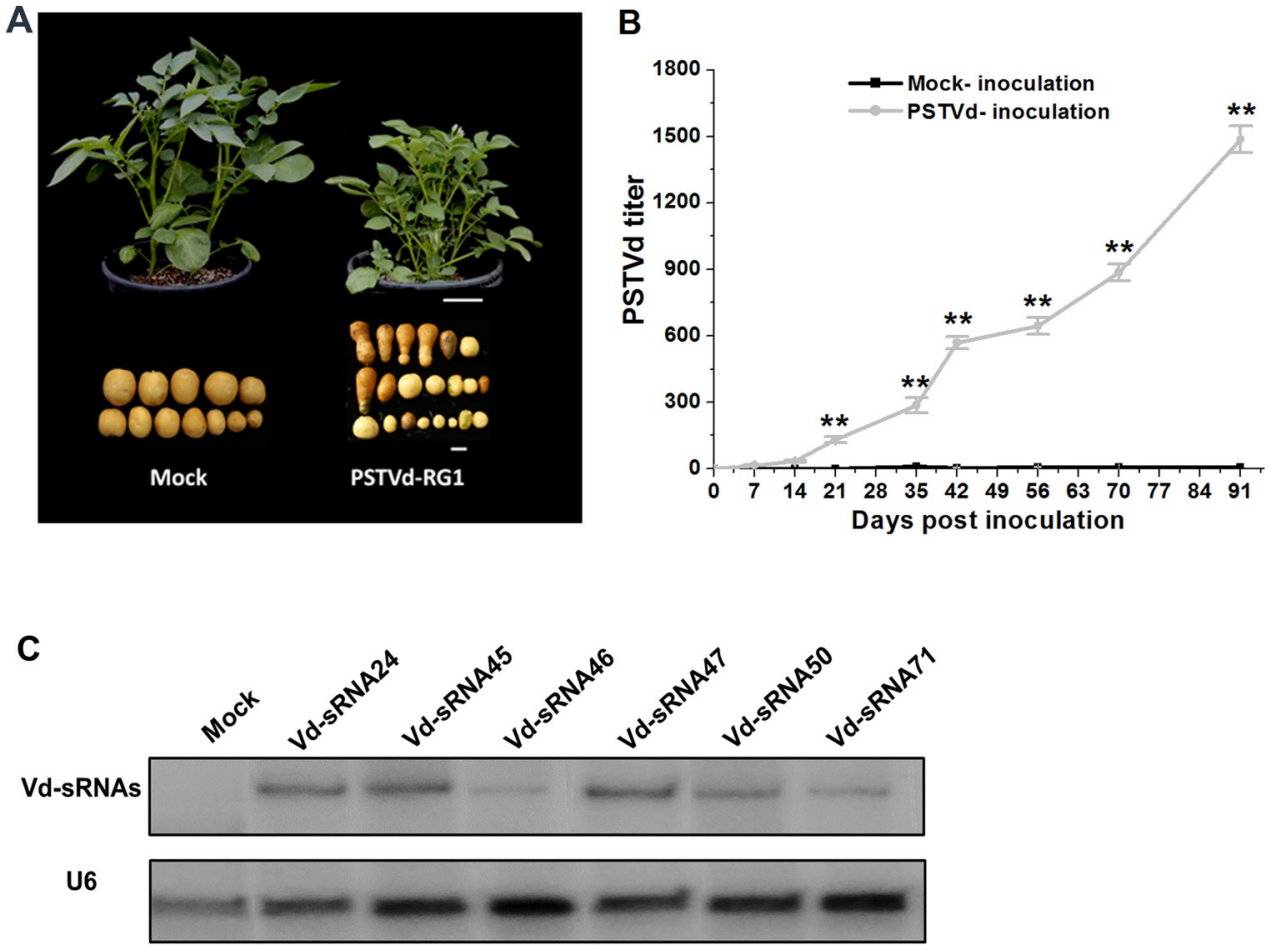
Symptoms associated with PSTVd-RG1 infection in potato. (A) Abnormal phenotype induced by PSTVd infection of potato cv. Atlantic at 60 dpi. Scale bar = 10 cm in whole plant or = 2 cm in tuber image. (B) Accumulation of PSTVd-RG1 progeny between 7–91 days post inoculation (dpi). Total RNA extracted from mock (black line) or PSTVd RG1-inoculated (grey line) potato plants was used to monitor the PSTVd titer using RT-qPCR. (C) Accumulation of small RNAs derived from the VM region of PSTVd-RG1 at 91 dpi. Small RNA extracted from mock inoculated or PSTVd-infected potato plants were analyzed by northern blot analysis using U6 RNA as an internal control.

The RG1 strain of PSTVd (PSTVd-RG1) was originally isolated from tomato, where severe disease phenotypes appears in sensitive cultivars upon infection by this strain of viroid [23]. Because the biological consequences of PSTVd-RG1 infection on potato were unknown, it was necessary to demonstrate the ability of the viroid to cause diseases in this host before investigating a possible role of viroid sRNAs-derived RNA silencing in symptom induction. We therefore inoculated young potato plantlets (cultivar Atlantic) with infectious RNA transcripts of PSTVd-RG1. The treated plantlets were transferred to a net house, and allowed to progress to maturity under natural light conditions. As shown in Fig 1A, PSTVd-infected plants were highly branched and stunted, with their leaves being upright, and slightly rugose. Tubers from infected plants were reduced in size, elongated, and spindle or dumbbell-shaped rather than being round.

Total RNA was extracted from young leaf tissue collected at various times up to 91 dpi and analyzed for the presence of both full-length viroid progeny (Fig 1B) and sRNAs derived from the VMR of PSTVd-RG1 (Fig 1C). PSTVd-RG1 progeny began to appear in the upper portions of inoculated plants between 14 and 21 dpi, and small RNAs from the upper portion of the VMR were clearly detectable in leaf tissue collected at 91 dpi. These two factors, the severity of plant reaction to infection by PSTVd-RG1 and evidence for vigorous levels of viroid replication, indicated that the combination of PSTVd-RG1 and potato cv. Atlantic is well-suited to studies of viroid pathogenicity.

### A potential target gene of VM-derived sRNAs is down-regulated in PSTVd-infected potato plants with distinctive vegetative phenotypes

**Fig 2:**
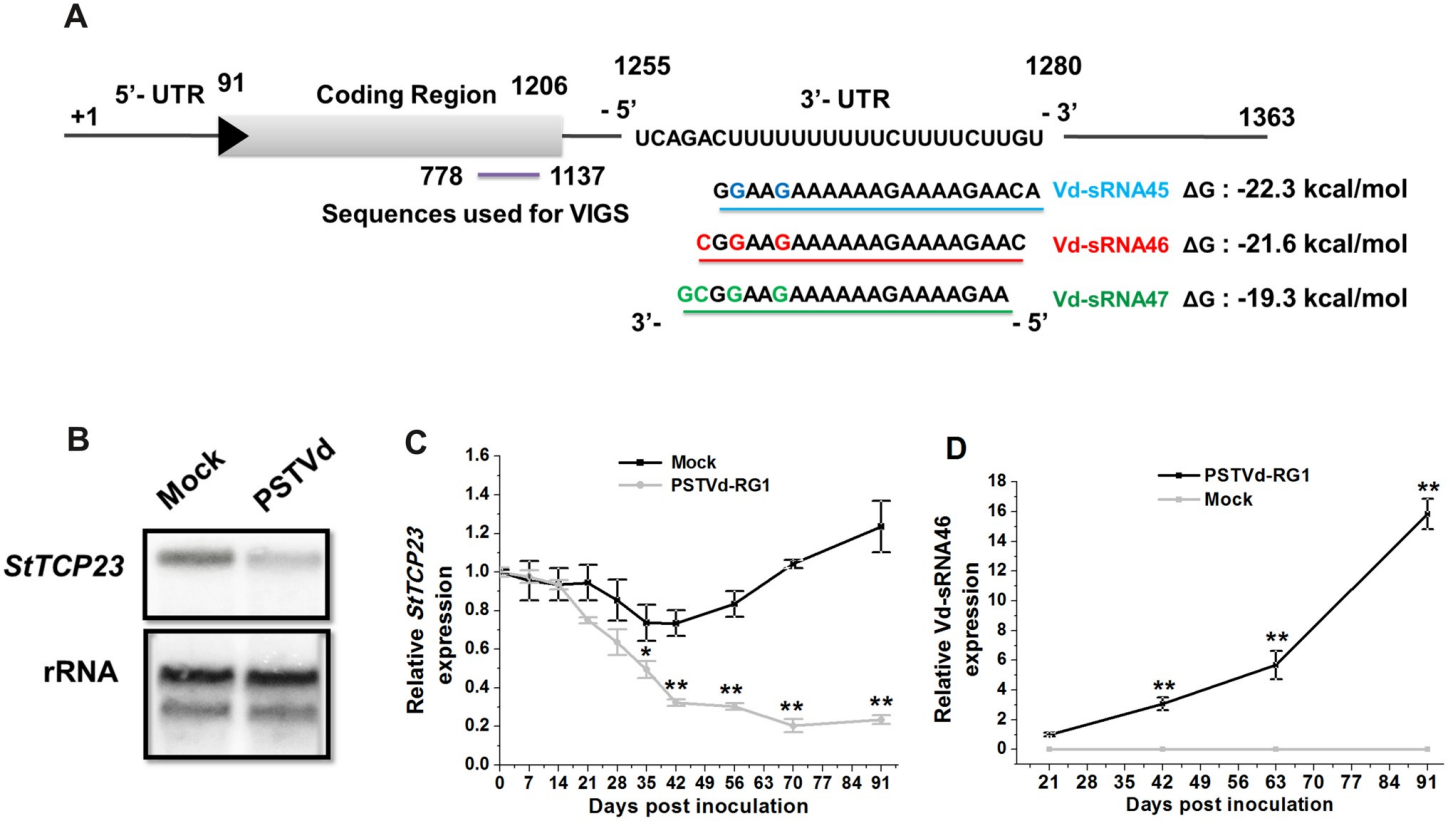
Targeting of *StTCP23* mRNA for silencing by small interfering (si) RNAs derived from PSTVd-RG1. (A) Schematic diagram of the mRNA encoding *StTCP23*. Complementarity between a target sequence located in the 3ʹ UTR and small RNAs derived from the PSTVd-RG1 genome and beginning at nt position 45 (blue line), 46 (red line), or 47 (green line) are shown. The PairFold online tool was used to predict the minimum free energy of the resulting RNA duplexes. Purple line, portion of the *StTCP23* coding sequence used for VIGS. (B, C) Effects of PSTVd-RG1 infection on *StTCP23* mRNA levels at different times post inoculation. Panel B, northern blot analysis of total leaf RNA extracted at 91 dpi, rRNA was used an internal loading control. Panel C, RT-qPCR analysis of the same series of RNA samples used to monitor PSTVd-RG1 replication (see Fig 1B). (D) Vd-sRNA46 expression profile in different development stage of PSTVd-RG1-infected plants.

To identify genes potentially targeted by PSTVd-derived sRNAs in potato, we used the web-based psRNATarget tool to search the potato transcriptome for sequences complementary to any 21-nt sequence segments of the PSTVd VM region. Results presented in Fig 2A show that sRNAs of 21-nt beginning at positions 45, 46 and 47 of the PSTVd-RG1 genome have the potential to target the 3ʹ UTR of a transcript which encodes a TCP transcription factor (*StTCP23*, *Solanum tuberosum* PGSC Acc. PGSC0003DMT400008728).

Northern blot hybridization analysis of RNA samples extracted at 91 dpi revealed a clear decrease in *StTCP23* transcript levels in infected plants (Fig 2B), and the results of RT-qPCR analysis of RNAs collected at various times during the assay provided further evidence for an inverse relationship between PSTVd-derived vd-sRNA46 and *StTCP23* transcript levels (Fig 2C and 2D).

### Virus-induced gene silencing of the *StTCP23* induces a PSTVd-like phenotypes in potato

In order to verify the role of altered *StTCP23* transcript abundance in the induction of PSTVd-like phenotypes, virus-induced gene silencing was used to suppress the expression of *StTCP23* in potato, and the phenotypes of the resulting virus-infected plants were monitored. As described in the Materials and Methods, a fragment from the coding region of the *StTCP23* gene, approximately 360-nt in length, was inserted into the pTRV2 vector (pTRV2: *StTCP23*). pTRV1 (TRV-RNA1) and pTRV2: *StTCP23* were then transformed into *Agrobacterium tumefaciens* and used to Agro-infiltrate potato seedlings. A fragment of the *phytoene desaturase* (*PDS*) gene, also inserted into the pTRV2 vector (pTRV2: *PDS*), served as a visual control to indicate successful viral infection, and negative control for this experiment was the Agro-infiltration of potato seedlings with an empty pTRV2 vector (pTRV2: EV).

**Fig 3:**
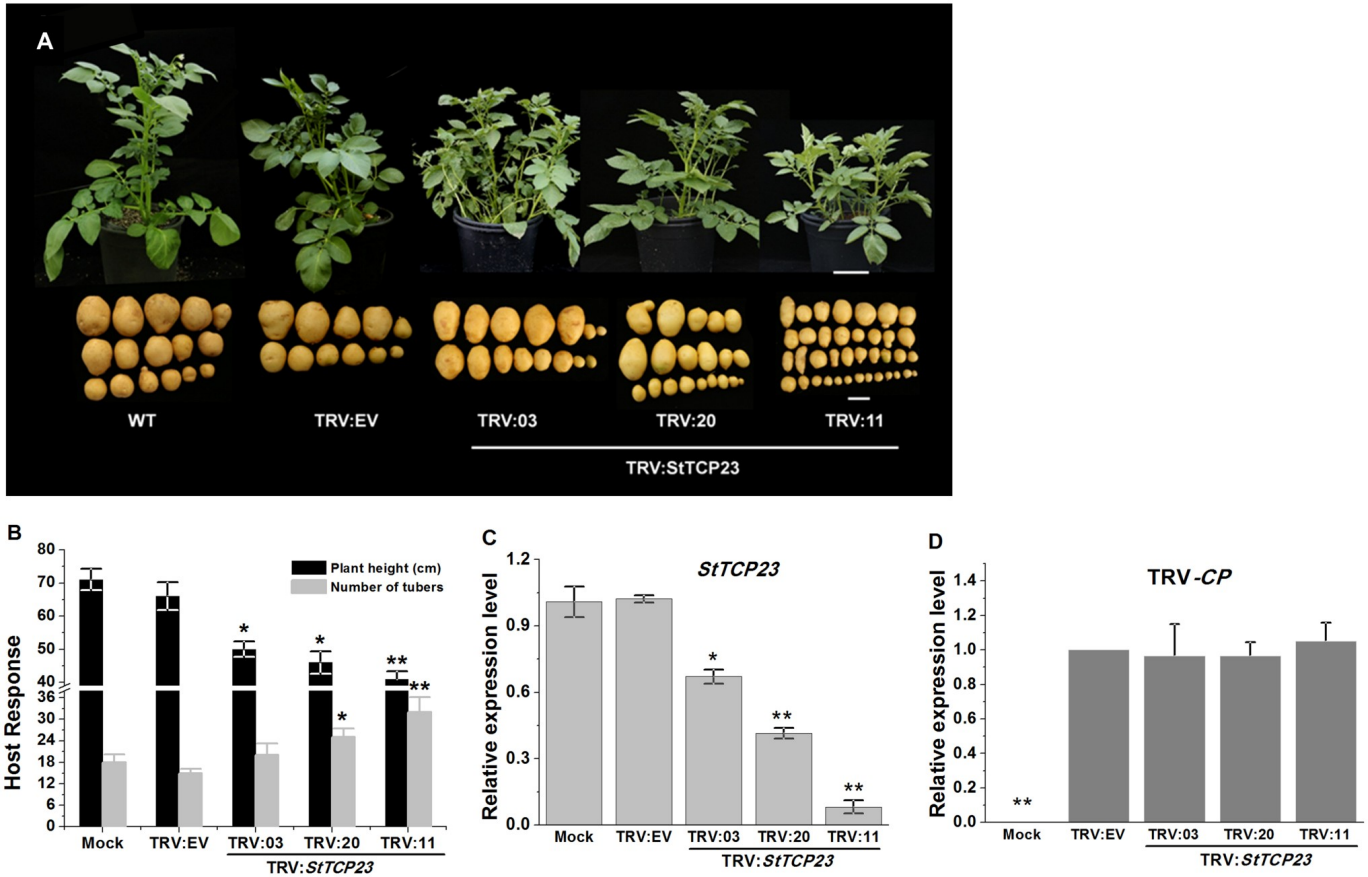
Virus-induced silencing of *StTCP23*. (A) Morphology of TRV-infected potato plants in which *StTCP23* expression has been suppressed by VIGS. Scale bar = 10 cm (whole plants) or 2 cm (tubers). (B, C) comparison of plant heights and number of tubers (B) and *StTCP23* mRNA expression levels (C) in TRV-infected and mock inoculated plants. (D) TRV capsid protein (CP)-coding gene expression levels in *StTCP23*-silenced and TRV empty vector-treated potato plants as measured by RT-qPCR.

Approximately two months post-inoculation, the plants in which *StTCP23* was the target of VIGS exhibited obvious abnormalities when compared to control plants. The pTRV2: *StTCP23* plants were stunted, and their leaves were twisted. Three months post-inoculation, the tubers produced by pTRV2: *StTCP23* plants were small and spindle-shaped, phenotypically very similar to those tubers produced by PSTVd-infected plants (Fig 3A and 3B). Importantly, the abundance of the *StTCP23* transcript was reduced in the pTRV2: *StTCP23* plants that expressed disease-like phenotypes. The expression of *StTCP23* in silenced plants and TRV accumulation level were determined by RT-qPCR. The three most recently-emerged leaves from each of three replicate plants showing an abnormal phenotype at 30 dpi of VIGS were used for analysis. The RT-qPCR results showed that the expression level of *STCP23* in silenced plants was suppressed by 30% (TRV:03), 55% (TRV:20) or 90% (TRV:11) compared to those in control plants (Fig 3C) and that there were no significant differences in TRV accumulation levels (Fig 3D). These data supported a role for *StTCP23* in regulating overall potato development.

### The 3ʹ UTR of *StTCP23* is targeted by PSTVd-derived sRNAs in a transient expression assay

To directly demonstrate that the predicted sRNA target sites in the 3ʹ UTR of the *StTCP23* transcript was a genuine target of vd-sRNA-directed silencing, a transient sensor reporter gene system was used to test sRNA-directed mRNA degradation. The 71-nt 3ʹ UTR sequence of *StTCP23*, carrying the predicted sRNA target sites, was transcriptionally fused to the 3ʹ end of the GFP coding sequence in the pCAMBIA1300-35S-GFP vector. This GFP fusion construct was then either co-infiltrated with a transient artificial miRNA expression construct created in pGreen II 62-SK and designed to express vd-sRNA46 (named TR-amiR46 here) or the empty vector control into potato leaves. Five days post infiltration, potato leaves were visually assessed for GFP fluorescence with reduced GFP expression taken to indicate TR-amiR46-directed repression of the 3ʹ UTR of *StTCP23*.

**Fig 4:**
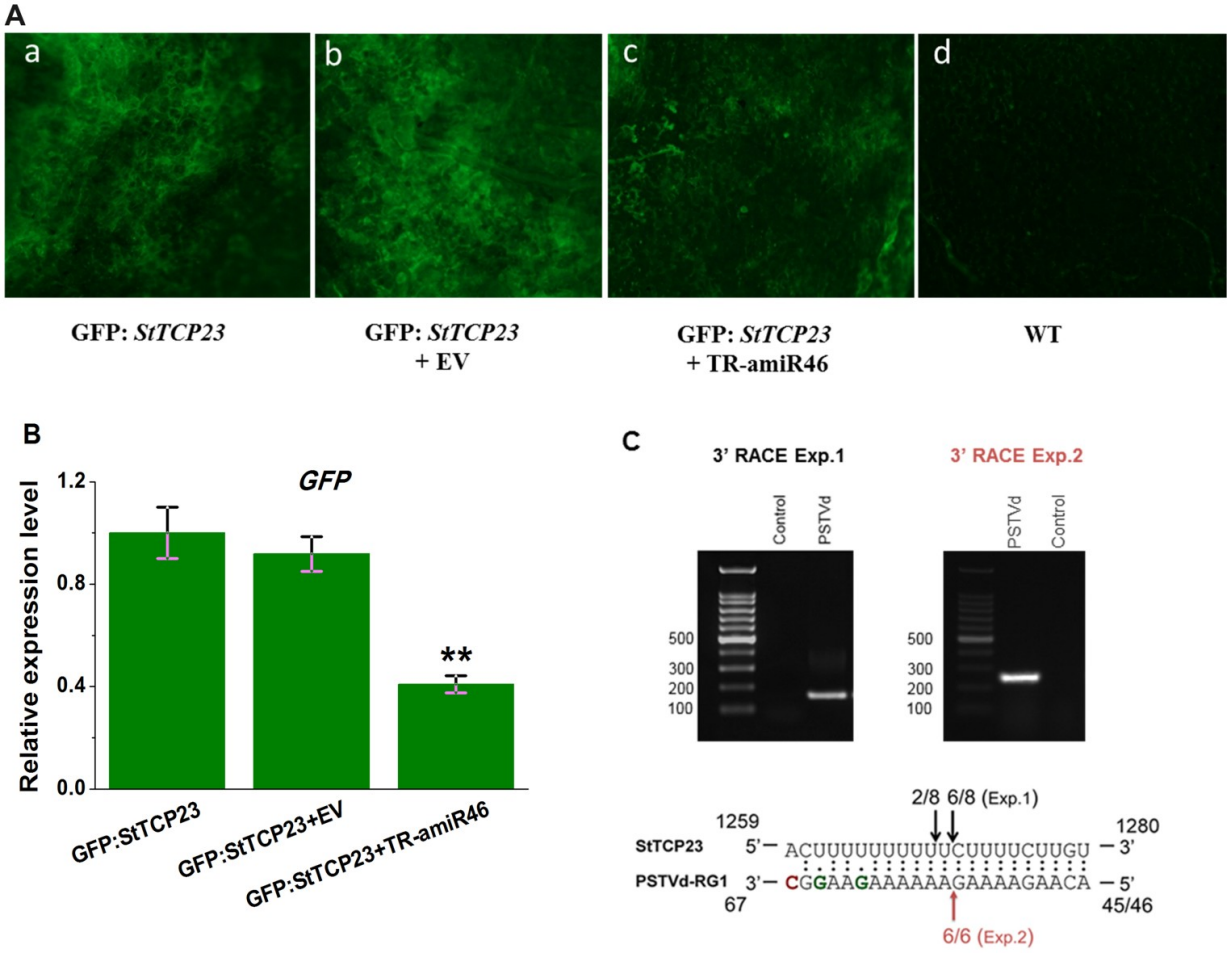
Predicted targeting of the *StTCP23* mRNA by vd-sRNA. (A) Potato leaves were agroinfiltrated with transient vector expressing (a) GFP: *StTCP23*, (b) GFP: *StTCP23* plus empty vector, (c) GFP: *StTCP23* plus TR-amiR46, (d) wild type control. At 5 dpi the leaves were photographed (4X) using a fluorescence microscope. (B) Potato leaves were agroinfiltrated using the same plasmid combinations as in (A). At 5 dpi relative GFP expression was quantified by RT-qPCR. (C) Nested PCR products obtained from 3′ RACE Exp.1 and 3′RACE Exp.2, using primers targeting the *StTCP23* mRNA were separated by 2.0% agarose gel electrophoresis. Predicted structure of the *StTCP23* mRNA/vd-sRNA duplex formed by siRNAs derived from the PSTVd-RG1. Arrows indicate the 3′ termini of *StTCP23* mRNA fragments isolated from the PSTVd-infected plants, as identified by 3′RACE Exp.1 and by 3′ RACE Exp.2, with the frequency of each termini shown.

As shown in Fig 4A, co-infiltration of the amiR46 vector together with the GFP: *StTCP23* 3ʹ UTR vector showed less GFP fluorescence than either the target GFP fusion construct alone, or following its co-infiltration with the empty vector. The difference in GFP expression was confirmed by RT-qPCR analysis, which showed that leaves co-infiltrated with the amiR46 vector contained less than 60% as much GFP mRNA as leaves infiltrated with the target GFP construct alone or when co-infiltrated with empty vector (Fig 4B). This result is consistent with the 3ʹ UTR sequence of the *StTCP23* gene being a bona fide target of TR-amiR46-directed RNA silencing.

### *StTCP23* mRNA undergoes sRNA-directed cleavage

To verify the targeting of *StTCP23* by PSTVd VMR-derived sRNAs, we next performed 3ʹ RLM RACE to detect any mRNA cleavage sites based on the methodology outlined in Adkar-Purushothama et al.[24] and Zuber et al.[25]. In brief, 3ʹ blocked adaptors were ligated to total RNA isolated from PSTVd-infected plants, and the ligation product was then used for cDNA synthesis with an adapter-specific reverse primer followed by product amplification by nested PCR (S1 Table). Two independent experiments were performed using different adaptors, rApp/CTGTAGGCACCATCAAT–NH 2 for 3ʹ RACE Experiment.1 and /5rApp/CTGACNNNNNNNNNNNNNNNTGGAATTCTCGGGTGCCAAGGC/3ddC/ for 3ʹ RACE Experiment. 2, which both gave distinct PCR product as shown in Fig 4C.

Sequences of the 3ʹ RLM RACE PCR clones were aligned with the *StTCP23* mRNA sequence to identify their 3ʹ termini. In 3ʹ RACE Exp.1, six out of eight *StTCP23*-matching clones from PSTVd- infected plants had a 3ʹ nucleotide corresponding to the predicted cleavage site between positions 10 and 11 of PSTVd VMd-sRNA45, and the remaining two *StTCP23*-matching clones shows cleavage sites corresponding to positions 10 and 11 for VM-sRNA46. Then in 3ʹ RACE Exp.2, all six analyzed *StTCP23* transcripts obtained for PSTVd-inoculated plants had 3ʹ termini identical to the predicted cleavage site between positions 10 and 11 of PSTVd VMd-sRNA45 (Fig 4C). No PCR amplification products were obtained when a similar experiment was performed using RNA extracted from uninfected wild type potato plants. These results therefore indicated sequence specific cleavage of the *StTCP23* mRNA at the predicted 3ʹ UTR site by PSTVd VMR-derived sRNAs, specifically the sRNAs starting at nt positions 45 and 46 of the PSTVd genome.

Cleavage at these two sites was consistent with the relatively strong sequence complementarity of sRNA45 and sRNA46, with the *StTCP23* target sequence compared with other potential VMR-derived sRNAs such as sRNA47 (Fig 2A). The detection of more frequent cleavage corresponding to sRNA45 than to sRNA46 could be due to the stronger target binding stability by sRNA45. In addition, the sRNA45 has a relatively weak 5ʹ A:U base-pairing in the precursor dsRNA duplex, a feature that favors argonaute loading compared to the stronger and less favored, 5ʹ C:G base-pairing of the sRNA46 precursor duplex.

A RT-qPCR analysis of the *StTCP23* mRNA sequences around the predicted vd-sRNA-binding site provided further evidence of vd-sRNA-mediated cleavage (S1A Fig). When oligo-dT primer was used for reverse transcription (RT), the downstream region gave much higher levels of amplification (PCR3) than the upstream (PCR2) and the region spanning the binding site (PCR1) in the PSTVd-infected and amiR46 plants but not in the uninfected plant. This indicated that the mRNA was cleaved at the binding site giving rise to poly(A)-containing 3ʹ vd-sRNA cleavage product. When using random hexamer RT primer, both the downstream and upstream regions showed higher levels of amplification than the cross-binding site region, indicating vd-sRNA-mediated cleavage and the existence of up and downstream cleavage fragments (S1B Fig).

### Expression of PSTVd VMR sRNAs as artificial miRNAs induces PSTVd-like symptom development in potato

**Fig 5:**
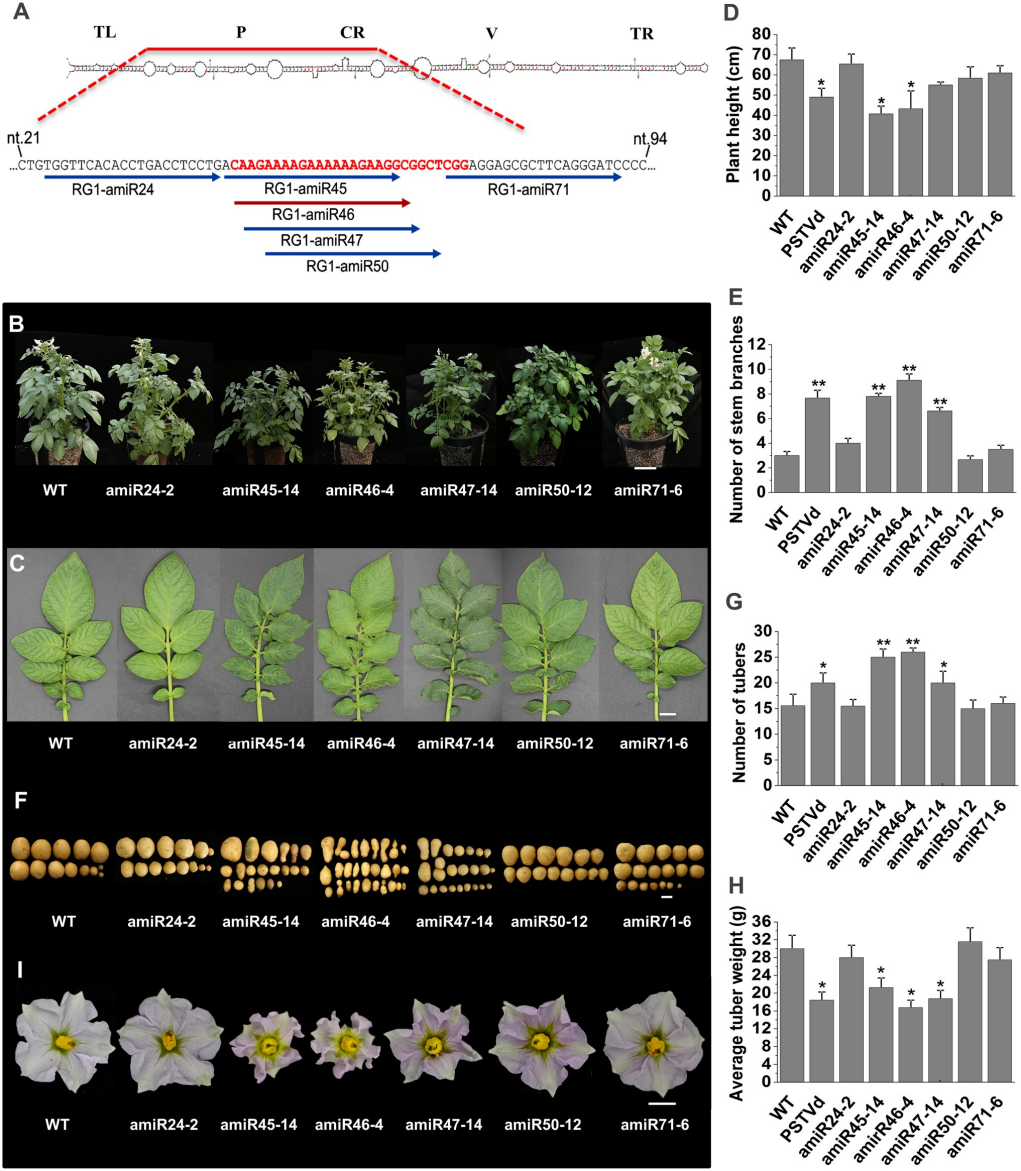
Evaluation of transgenic lines expressing six PSTVd amiRNA vectors. (A) Rod-like native structure of PSTVd-RG1. The sequence of the upper portion of the V(irulence) M(odulating) region within the Pathogenicity domain [P] is highlighted in red, and sequences of four amiRNAs derived from the upper portion of the VM region as well as two other amiRNAs derived from flanking sequences are shown below. (B) Whole plant phenotypes. Scale bars = 10 cm. (C) Leaf phenotypes. Scale bars = 1 cm. (D) Comparison of average plant height and (E) number of stem branches for amiRNA-expressing and control plants. (F) Tuber phenotypes. Scale bars = 3 cm. (G) Comparison of tuber numbers and (H) average tuber weights for amiRNA-expressing and control plants. (I) Flower phenotypes for amiRNA-expressing and control plants.

To further examine the role of PSTVd VMR-derived sRNAs in disease symptom development, we transformed potato plants with six amiRNA expression vectors designed to generate 21-nt small RNAs corresponding to VM region and non-VM regions. Among the six constructs, four were designed to generate mature 21-nt sRNAs corresponding to VMR sequences of PSTVd strain RG1, starting at genome nt positions, 45, 46, 47 and 50 (amiRNAs amiR45, amiR46, amiR47 and amiR50, respectively) (Fig 5A). It is worth noting that the lethal PSTVd strains RG1, and KF440-2 show sequence differentiation at nucleotides 46 (C) and 47 (A) compared to the intermediate and mild strains, which have nucleotides G and C at nt. 46 and 47, respectively. Lethal strain AS1 shows partially sequence differentiation at nucleotides 46 (C) and 47 (U). These nucleotide variations in the intermediate and mild strains would result in two mismatches at the 5ʹ region of sRNA between the VMR sRNAs and *StTCP23* mRNA (S2 Fig). This would be expected to dramatically reduce the target binding and cleavage efficiency as mismatches at the 3ʹ region of sRNA, particularly at the “seed region” (nt. 2–8), are highly disruptive to target RNA binding. The remaining two constructs (amiR24 and amiR71) were designed to represent sRNAs derived from non-VMR sequences either upstream or downstream from sequence that immediately flanks the VMR of PSTVd-RG1, and further, these two amiRNAs were determined to have no sequence complementarity to any known *solanum tuberosum* target genes [19]. The number of transgenic lines randomly selected for phenotypic analysis is listed in Table 1, and the typical phenotypes and the frequency of phenotype expression are further summarized in Table 1 and Fig 5.

**Table 1:** Proportion of randomly selected primary potato cv. Atlantic transformant lines expressing developmental abnormalities.

**(A) Foliage symptoms**				
	None	Mild	Moderate	Severe
**amiR24**	25/25^a^	0	0	0
**amiR45**	0/27	3/27	14/27	10/27
**amiR46**	0/24	4/24	9/24	11/24
**amiR47**	0/19	7/19	10/19	2/19
**amiR50**	10/12	2/12	0	0
**amiR71**	16/16	0	0	0
**(B) Tuber shape**				
**amiRNA vectors**	**Round**		**Elongated/spindle-shaped**	
**amiR24**	100^b^		0	
**amiR45**	9		91	
**amiR46**	10		90	
**amiR47**	33		67	
**amiR50**	96		4	
**amiR71**	100		0	

^a^ Plants showing symptoms/total plants.

^b^ Data expressed as percentages.

None of the plants expressing amiR24 or amiR71 displayed abnormal phenotypes with both their above and below ground tissues developing normally (Fig 5). Of the twelve amiR50 lines examined, only two lines showed mild phenotypic abnormalities. In contrast, the *in planta* expression of the other three VMR-specific amiRNAs, amiR45, amiR46 or amiR47, all induced phenotypes similar to those that result from PSTVd infection of potato. Abnormal foliar phenotypes, from mild to severe, occurred in all amiR45, amiR46 and amiR47 transformant lines assessed (Table 1A). Mild foliar phenotypes included a reduction in plant height and a slight twisting of leaves without a change in the pattern of branching. Intermediate phenotypes included stunting, increased stem branching, and leaf twisting; effects previously observed in PSTVd-infected potato (Fig 5B and 5C). Ten of the 27 amiR45 lines, eleven of the 24 amiR46 lines, and two of the 19 amiR47 lines displayed severe PSTVd-like disease symptoms, namely, severely stunted growth with significant increases in the numbers of main and lateral stems, loss of apical dominance, and a “*bunchy top*” appearance caused by shortened internode length (S3A Fig). The severe foliar symptoms also included smaller sized leaves that displayed downward curling at their margins, and a more upright growth habit. Overall, the average height of amiR45, amiR46 and amiR47 transformants was reduced by 26.8, 24.35 and 12.45 cm (Fig 5D), and the average number of stem branches was increased by 4.8, 6.1 and 3.6, respectively (Fig 5E).

### Transformant lines expressing VMR-derived amiRNAs display PSTVd-like tuber and floral phenotypes

To assess tuber shape, tubers were harvested after 4 months of growth in a net house. Eighteen individual transformant lines containing each amiRNA plant expression vector were investigated. For plants expressing amiR24 and amiR71, only normal tubers (i.e., non-spindle shaped) were observed. For amiR50 expressing plants, only 4% of tubers showed elongated or spindle shaped. In contrast, 91% of amiR45, 90% of amiR46, and 67% of amiR47 tubers displayed the elongated or spindle shape characteristic of tubers from PSTVd-infected plants (Table 1B). The plants expressing amiR45 and amiR46, in particular, produced more stolons than either the WT control plants or the other transformed populations that stemmed from the *in planta* expression of the other 4 amiRNA plant expression vectors. The promotion of stolon formation led to the formation of spindle-shaped, elongated and knobby tubers similar in shape to tubers of PSTVd-infected plants. A large percentage of such tubers were small in size, a feature not seen in control plants (Fig 5F). Statistical analysis revealed that amiR45, amiR46, and amiR47 expressing plant lines generated higher numbers of tubers than either wild-type plants or those expressing any of the other three amiRNAs, but that these tubers were reduced in size with lower total weights (Fig 5G and 5H). Further, the tubers that formed on amiR24, amiR71 or amiR50 plants, were similar in weight to those of wild-type plants. In particular, the number of tubers harvested from one pot of amiR45 and amiR46 lines was elevated by 1.5-fold compared to either wild-type control plants or those expressing other amiRNAs. However, in spite of this increased tuber number, the weight of each amiR45 and amiR46 tuber was reduced by 2-fold. As shown in S3B Fig, the above ground portions of some amiR45 and amiR46 plants matured early, often requiring only 2 months from seedling germination to senescence and eventual death compared to the 4–5 months life cycle typical of wild-type control plants. In addition, the progression of flower development and tuber formation was also accelerated in these amiR45 and amiR46 transformant lines.

PSTVd infection also has been reported to have a negative effect on the sexual reproduction of the infected host plant. Specifically, viroid transmission in true potato seed as well as a 50% reduction to pollen viability has been reported for PSTVd-infected tomato [26, 27]. We therefore investigated flower development in the amiRNA transformants. Almost all plants expressing either amiR45 or amiR46 flowered later and produced fewer flowers than either the wild-type control plants or transformants that expressed amiR24 or amiR71. Several lines also exhibited abnormal floral structures such as shriveled petals (Fig 5I) or deformed anthers (S3C Fig). Furthermore, the amiR45 and amiR46 transformant lines also produced reduced amounts of pollen compared to either wild-type plants or the other amiRNA transformant lines. The pollen produced by amiR45 and amiR46 plants also had reduced viability and low fertility. Plants expressing amiR47 or amiR50 vector also exhibited reduced pollen viability compared to wild-type plants or the amiR24 and amiR71 transformant lines (S3D Fig).

### The severity of the developmental phenotypes expressed by amiRNA lines is correlated with amiRNA abundance and repression of *StTCP23* expression

**Fig 6:**
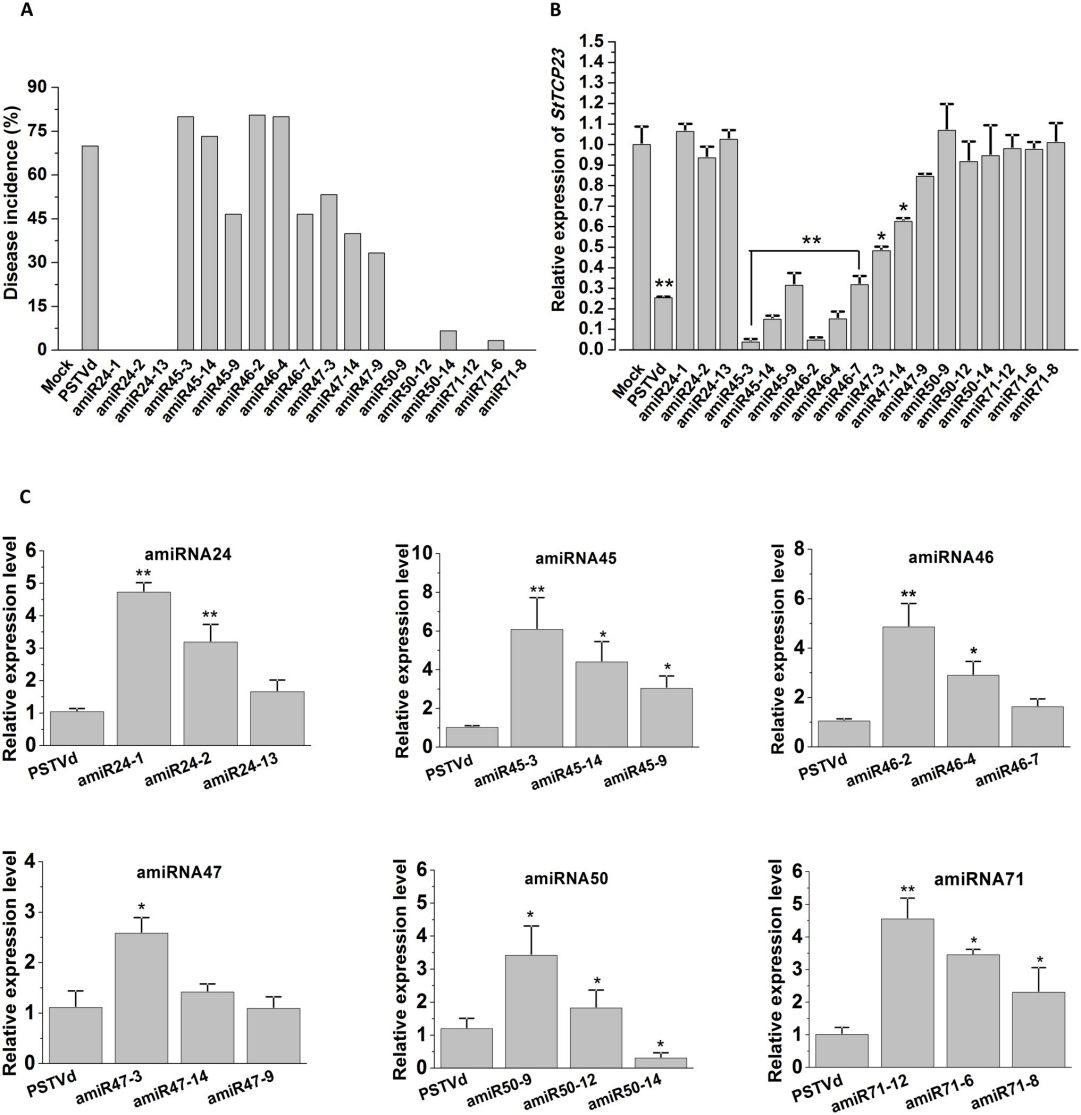
Variation in amiRNA and *StTCP23* expression among different transgenic potato lines expressing amiRNAs. (A) Disease severity index of amiRNA transgenic lines. Mean values (n = 18) are shown. (B) RT-qPCR quantitation of PSTVd sRNA levels in transgenic lines. In each panel, the leftmost bar is a control derived from PSTVd-RG1-infected non-transgenic plants. From left to right, the remaining three bars are values from transgenic plants expressing severe, intermediate, or mild symptoms. (C) Relative *StTCP23* mRNA in amiRNA transgenic lines expressing severe, medium, or mild symptoms.

The abundance of each amiRNA sRNA in the leaf tissues of individual amiRNA lines were estimated via RT-qPCR. For each amiRNA construct, three independent lines that displayed a differing degree of phenotype severity (Fig 6A) and showed altered amiRNA abundance via RT-qPCR (Fig 6B) were again analyzed via RT-qPCR to determine *StTCP23* expression levels (Fig 6C). However, we first attempted to establish the spatial expression pattern of *StTCP23* in healthy wild-type potato plants. According to FPKM data (fragments per kilobase of transcript per million mapped reads) data obtained from the PGSC database (http://solanaceae.plantbiology.msu.edu/pgsc_download.shtml), *StTCP23* is widely expressing in all tissues, especially expressed highly in DM1-3 petiole, shoot and leaf, it was also expressed highly in RH petiole, stolon, stem, leaf and flowers (S4A and S4B Fig). Using the RT-qPCR approach, the *StTCP23* gene was determined to be highly expressed in nodes, stolon, stem and dormant tubers, but was also expressed at a readily detectable level in the leaf (S4C Fig), indicating that *StTCP23* may play an important role in stem branching, tuber development and leaf morphologies. In order to normalize the test point, especially considering of the small RNAs and PSTVd transmission and accumulation activity in plant tissues, we choose leaf tissue as the sampling point in further *StTCP23* detection.

As shown in Fig 6C, the expression of *StTCP23* was significantly down-regulated in PSTVd-infected plants and in amiR45, amiR46 and amiR47 transformant lines. In addition, the degree of *StTCP23* down-regulation showed a strong inverse correlation with the abundance of each amiRNA that accumulated in each amiRNA transformant line (Fig 6B and 6C). RT-qPCR also revealed that the overall level of *StTCP23* down-regulation was stronger in amiR45 and amiR46 lines than in amiR47 lines, which is consistent with the generally more severe phenotypes displayed by the amiR45 and amiR46 transformant lines than by the transformant lines expressing the amiR47 sRNA. Furthermore, this result was also consistent with the amiR45 and amiR46 sRNAs having a more extensive sequence complementarity to the *StTCP23* mRNA than the amiR47 sRNA (Fig 2A). The data presented in Fig 6 also reveals strong correlation between amiRNA accumulation and the severity of PSTVd-like phenotypes displayed by the amiR45, amiR46 and amiR47-expressing plant lines. Plant lines, amiR45-3 and amiR46-2, which exhibited the most severe phenotypes (Fig 6A) among the three tested lines of each amiRNA population during the three-month assessment period, contained the highest amiRNA level and had the greatest reduction (approximately 20-fold) in *StTCP23* expression. Plant lines, amiR45-14 and amiR46-4, which displayed intermediate phenotypes, showed a lower level of amiRNA accumulation and *StTCP23* down-regulation (5 to 10-fold). No significant down-regulation of *StTCP23* expression was detected in the plant lines that expressed any of the other amiRNA constructs (Fig 6B and 6C), a finding that was consistent with the general lack of PSTVd-like phenotypes in these plants. Taken together, our results showed a clear correlation between the abundance of PSTVd VMR-derived amiRNAs, the degree of *StTCP23* down-regulation, and the severity of the PSTVd-like phenotypes displayed by the amiRNA lines. These results indicate that PSTVd VMR-derived sRNAs target the *StTCP23* gene in potato to induce disease symptom development.

### Survey for other potential target genes for PSTVd amiRNAs

To investigate whether other potato genes might be targeted by the VMR-derived amiRNAs thereby contributing to the disease phenotypes observed in the transgenic plants, we searched the potato genome for potential target genes using the software http://plantgrn.noble.org/v1_psRNATarget/ for target gene prediction and https://www.uniprot.org/blast/ for protein blast. Expression of the putative target genes was then analyzed using RT-qPCR in amiRNA lines as well as in PSTVd-infected plants. Results from these analyses are listed in S1 File.

*StTCP23* was the top-ranking target gene for amiRNA46 based on the stability of amiRNA::target gene duplex. For amiRNA45, *StTCP23* ranked second based on duplex stability, but the first and third ranking genes as well as most of the remaining genes all contained a G:U wobble base pair at one of the central nucleotides, which would negatively affect the target cleavage efficiency (base pairing at nucleotides 10 and 11 of sRNA is critical for target RNA cleavage). Thus, *StTCP23* was predicted to be the best target for both amiRNA45 and amiRNA46. Consistent with the sequence alignment results, *StTCP23* showed the strongest and most consistent down-regulation in both the amiRNA lines and PSTVd-infected plants (S5 and S6 Figs). Additionally, some of the other down-regulated targets are not well-defined or annotated, and some are involved in general cellular functions of plants, with few being directly related to plant growth and development. Fewer potential target genes were identified for amiRNA47 than amiRNA45 and amiRNA46. Five of these putative target genes for amiRNA47 showed downregulation in PSTVd-infected plants, of which three, including *StTCP23*, membrane-anchored ubiquitin-fold protein and glutaredoxin-C9 coding genes were also downregulated in the amiRNA47 plants. These additional putative target genes merit further investigation in future studies, but the relatively weak phenotypes of the amiRNA47 plants as compared with those expressing amiRNA45 or amiRNA46 suggested that these other genes do not play a major role in development of PSTVd- induced phenotypes in potato.

Three putative target genes were predicted for amiRNA50, but only one showed downregulation in PSTVd- infected and amiRNA50 plants. Again, the weak phenotypes of the amiRNA50 transgenic population indicated that this putative target was unlikely to be a primary target for PSTVd disease induction. Only one and two putative target genes were predicted for amiRNA24 and amiRNA71, respectively, but the central portions of the respective amiRNA::target duplexes contained multiple G:U base pairs, which is likely to result in weak target RNA cleavage. This is consistent with the observed lack of phenotypes in the amiRNA24 and amiRNA71 plants. Thus, this target gene survey confirmed the status of *StTCP23* as the top candidate gene for PSTVd sRNA-induced RNA silencing in potato. This result also supports the dominant role of the interaction between Vd-sRNA45/46 with *StTCP23* in PSTVd-induced disease symptom development.

### Similar effects on tuber formation and sprouting behavior in amiRNA transgenic and PSTVd-infected plants

Seed potatoes infected by PSTVd routinely show delayed plant emergence or, reduced rate of emergence, in the next generation [28]. Furthermore, the germination efficiency of PSTVd-infected tomato seed is significantly lower (53%) than non-infected seed (98%) [29]. We therefore investigated the sprouting and emergence performance of tubers harvested from healthy plants, PSTVd-infected plants, and plants expressing either the amiR46 or amiR71 vector.

**Fig 7:**
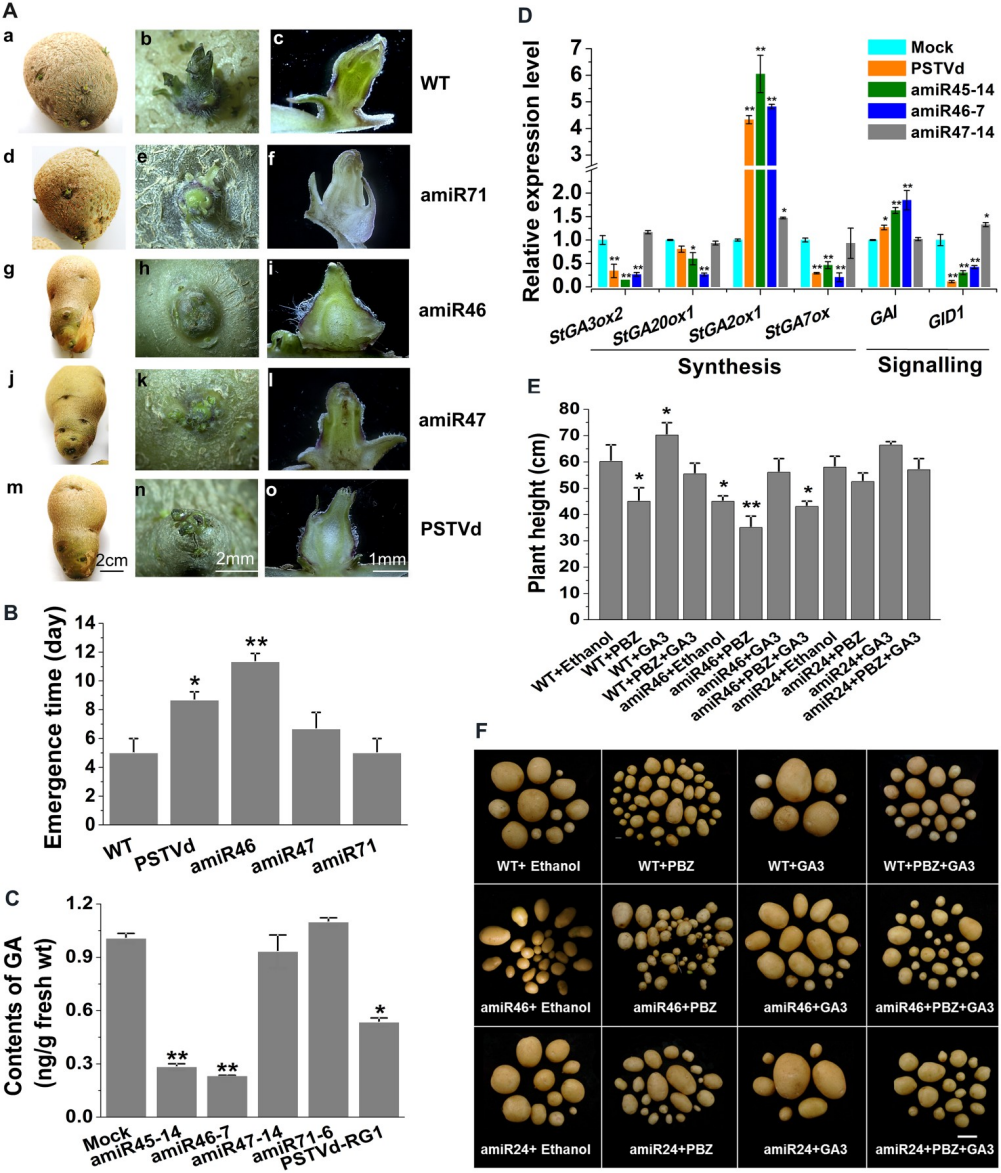
Regulation of the GA pathway by amiRNA46 and *St*TCP23. (A) Sprouting behavior of tuber discs prepared from amiRNA transgenic and control potato lines. (a, b, c), mock inoculated plants; (d, e, f), transgenic plants expressing amiR71; (g, h, i), transgenic plants expressing amiR46; (j, k, l), transgenic plants expressing amiR47; (m, n, o), PSTVd inoculated non-transgenic plants. (a, d, g, j, m), sprouting behavior of whole tubers, scale bar = 2 cm; (b, e, h, k, n), sprouting behavior of individual buds, scale bar = 2 mm; and (c, f, i, l, o), cross sections of individual sprouting buds under the scanning electron microscope, scale bar = 1 mm. (B) Delayed emergence of new stems in transgenic plants expressing amiR46, amiR47 and PSTVd-inoculated non-transgenic plants as compared with transgenic plants expressing amiR71 and uninoculated non- transgenic control plants. (C) Comparison of endogenous GA_3_ levels in uninfected control, amiR45, amiR46, amiR47, amiR71, and PSTVd-infected non- transgenic plants. (D) RT-qPCR quantitation of selected genes involved in gibberellin metabolism. Total RNA was extracted from tuber harvested at bulking stage during potato tuber development. (E) Comparison of plant height for the different treatments. (F) Morphology of tubers collected from non-transgenic and amiR46, amiR24 transgenic plants after treatment with ethanol, PBZ, or GA_3_. Scale bars = 3 cm.

After storage for two months under natural light conditions, tubers started to sprout. The sprouting behavior of the amiR46 tubers was altered in terms of the number of sprouts produced per tuber. As shown in Fig 7A, the buds from the harvested amiR46 tubers also exhibited a low degree of germination and produced sprouts as the aerial tubers were developing. Tubers from the uninfected control plants showed a higher degree of sprouting during storage than either amiR46 or PSTVd-infected plants. Shoots were evenly distributed over the surface of healthy tubers with an average of 8–10 buds per tuber that produced sprouts of approximately 6.0 mm in length after storage for 10 weeks at room temperature. Tubers from amiR46 lines showed a lower level of sprouting during storage than the controls, and buds were distributed only around the navel. There were only 1–3 abnormal buds per tuber with an average shoot length of approximately 2.6 mm. New tubers also formed from sessile buds derived from mother tubers, resulting in protruding eyes and/or knob-like tubers. Moreover, in transverse sections of tubers from amiR46 and PSTVd-infected plants, axillary meristems appeared misshapen or developmentally disabled. In addition, outgrowth of lateral buds led to the production of knobby tubers, a condition not seen in control plants (Fig 7A). After the sprouted tubers were transferred to pots containing soil, young plantlets emerged from the bud eyes between 3–10 d after planting. Emergence time was significantly delayed in amiR46 and PSTVd-infected plants as compared with either the wild-type control or the amiR47 and amiR71 plant lines (Fig 7B).

### *StTCP23* interacts with the GA pathway to affect tuber development

The life cycle of a potato tuber is controlled by cycles of meristem activation and deactivation mediated via symplastic association and disassociation of the tuber apical bud [30–32]. Previous studies have shown that gibberellins are the most important hormonal regulators of bud outgrowth, and seed potato sprouting are also thought to be involved in stolon growth and tuber development [33–37]. *St*TCP23 is a class I TCP transcription factor whose closest paralogs in *Arabidopsis*, *At*TCP14 and *At*TCP15 (S7A Fig), mediate gibberellin-dependent activation of the cell cycle during germination [38, 39]. In order to assess the possible involvement of *St*TCP23 in the GA pathway during tuberization, we compared the GA contents of tubers collected from amiRNA transgenic and control plants. The resulting data revealed that GA levels were significantly lower in tubers from either amiR45 and amiR46 plant lines or from PSTVd-infected plants, as compared to the GA levels of amiR47 and amiR71 tubers or the tubers of uninfected Atlantic plants (Fig 7C). Consistent with this reduced GA level, RT-qPCR analysis showed that the expression levels of the GA biosynthesis-associated genes, *StGA3ox2*, *StGA20ox1* and *StGA7ox*, were all down-regulated in amiR45, amiR46 and PSTVd-infected plants. This down regulation coincided with the up-regulation of the GA degrading gene, *StGA2ox1* (Fig 7D).

DELLA proteins modulate multiple signaling pathways through physical interaction with transcription factors that include members of the TCP transcription factor family [40–42]. Previous studies have shown that *At*TCP15 and *At*TCP14 (the two *Arabidopsis* transcription factors exhibiting the highest homology to *St*TCP23) are able to interact with *At*GAI and *At*RGL2, two DELLA proteins involved in the regulation of germination in *Arabidopsis* [43–44]. RT-qPCR analysis revealed increased expression of *GAI* in amiR45, amiR46 and PSTVd-infected plants as compared to mock inoculated wild-type potato plants (Fig 7D). Decreased gibberellin content has also been reported to result in decreased expression of the gibberellin receptor gene, *GID1* [42, 45, 46]. Consistent with this finding, our data revealed that compared to mock inoculated wild-type potato plants, *StGID1* transcript levels were slightly, but significantly, down-regulated in the amiR45, amiR46 and PSTVd infected plants (Fig 7D). Taken together, these results suggested that *StTCP23* down-regulation in amiR45 and amiR46 plants as well as PSTVd-infected plants may reduce GA accumulation by changing the expression levels of genes encoding key enzymes in GA metabolism, thereby influencing bud growth.

In order to further explore the role of *St*TCP23 in mediating changes in GA levels during tuber development, we compared the effects of treating wild-type control plants as well as those expressing amiR46 or amiR24 with either ethanol, GA_3_, the GA_3_ inhibitor paclobutrazol (PBZ), or a combination of GA_3_ plus PBZ. PBZ-treatment of wild-type plants produced greater numbers of smaller sized tubers than control plants treated with ethanol alone (S7B and S7C Fig). Treated plants resulted in stunting and twisting. GA_3_ treatment restored the height of amiR46 plants to that of normal wild-type plants (Fig 7E). In addition, treatment of amiR46 plants with GA_3_ resulted in the formation of large, round tubers (Fig 7F). Furthermore, the application of GA_3_ to previously PBZ-treated plants, restored plant height and tuber number to levels observed in control plants treated with ethanol alone (Fig 7E, S7B and S7C Fig). Taken together, these results strongly suggested that *St*TCP23 positively regulates potato plant sprouting and tuber development via a GA-dependent mechanism.

## Discussion

Potato is the third largest food crop globally, surpassed only by rice and wheat. PSTVd infection has a significant impact on the yield and quality of potato, causing such characteristic disease symptoms as stunted growth, and the formation of small and spindle-shaped tubers. As a vegetatively propagated crop, the quality of seed potato tubers is extremely important to potato production. However, once infection is initiated, PSTVd is difficult to eliminate from infected tubers [47, 48], making it an extremely difficult pathogen to control.

The importance of the nucleotide sequence of its non-protein-coding genome in PSTVd disease symptom development has long been recognized. For instance, the nucleotide sequence of the virulence modulating region (VMR) located on the left side of its rod-like RNA structure is known to be important in directing PSTVd pathogenicity, with one to a few nucleotide changes leading to dramatic differences in disease severity [49, 50]. Small RNAs derived from the VMR region have been previously predicted and/or demonstrated to target different host genes for silencing in several plant species such as tomato and *Nicotiana*, and that this sRNA-induced host gene expression modulation has been proposed to account for the induction of PSTVd disease-like symptoms in infected plants [2, 19, 20, 24, 51–53]. However, the molecular events and/or pathways linking the modification of host gene expression to the disease symptom development remain to be identified. Furthermore, whether VM-derived sRNAs also target host genes in potato and whether sRNA-directed host gene silencing is responsible for the PSTVd disease symptoms observed in this species have remained unknown.

In the present study, we identified the transcript of *StTCP23*, a potato gene encoding TCP transcription factor, as a potential target for VMR-derived sRNA-directed expression regulation. Bioinformatic analysis revealed a high level of sequence complementarity between the 3ʹ UTR of *StTCP23* and the VMR sequence from nt 45 to 65 of the PSTVd-RG1 genome. Using a combination of northern blot and RT-qPCR analysis, we first demonstrated the accumulation of 21-nt sRNAs specific to the VMR in PSTVd-inoculated plants 3 months post inoculation. Furthermore, this accumulation of VMR-specific sRNAs was associated with decreased *StTCP23* transcript abundance and PSTVd disease symptom development. 3ʹ RLM RACE analysis of PSTVd-infected potato plants detected *StTCP23* cleavage products that aligned to the expected cleavage position within the region of *StTCP23* sequence homology to the PSTVd VMR. Also, virus-induced gene silencing of *StTCP23* mRNA, in the absence of PSTVd infection, resulted in PSTVd-like phenotypes in potato. Taken together, these results provide strong evidence that VMR-derived sRNAs direct mRNA cleavage-based silencing to repress the expression of *StTCP23* upon PSTVd infection.

To demonstrate that reduced *StTCP23* expression was specific for the VMR-derived sRNAs, and that this VMR-sRNA-directed expression repression was responsible for the appearance of PSTVd disease symptoms, we expressed a series of 21-nt artificial miRNAs having the same sequences as the VMR-derived sRNAs in transgenic potato. Expression of amiR45 and amiR46, corresponding to Vd-siRNA45 and Vd-sRNA46, resulted in both the down-regulation of the putative target gene, as well as the expression of phenotypes closely resembling those displayed by wild-type potato plants upon infected by PSTVd-RG1. Furthermore, some of the amiR46 transformant lines also matured, set tubers, and entered senescence, more rapidly than non-transformed potato plants. Lines expressing amiR47 or amiR50, corresponding to VMR sequences beginning at genome positions nt 47 and nt 50 respectively, also exhibited a similar array of PSTVd-like symptoms, albeit with reduced severity, and in a smaller proportion of the total transformant population. In contrast to the lines expressing VMR-derived amiRNAs, potato expressing amiR24 or amiR71 sRNA, sRNAs that correspond to the sequences flanking the VMR, were wild type-like in both plant growth and tuber formation and failed to display any readily observable phenotypes.

The relatively weak phenotypes associated with the in planta expression of the amiR47 sRNA, in comparison to the more severe phenotypes displayed by plant expressing amiR45 or amiR46, were likely due to the reduced number of perfectly matched or G:U wobble base-pairings in the amiR47::*StTCP23* duplex as compared to either the amiR45::*StTCP23* or amiR46::*StTCP23* duplex. Similarly, comparison of amiR50 with the 21-nt *StTCP23* target gene sequence reveals only 14 nts of perfect complementarity for the corresponding duplex. The predicted thermodynamic stability of the amiR50::*StTCP23* duplex (ΔG = -7.75 kcal/mol) was also much weaker than any of the corresponding duplexes formed between the *StTCP23* target gene and the amiR45 (ΔG = -22.3 kcal/mol), amiR46 (ΔG = -21.6 kcal/mol), or amiR47 (ΔG = -19.3 kcal/mol) sRNAs. This difference in thermodynamic stability likely accounts for the fact that weakest phenotypic effects were observed for the amiR50 transformant lines among the four VMR-derived amiRNA transformant line populations studied. BLAST searches of currently available *S*. *tuberosum* transcriptome data for sequences complementary to two non-VMR amiRNAs, amiR24 and amiR71, failed to identify any putative target transcripts of functional relevance, suggesting that neither sRNA contributes to PSTVd-induced host gene silencing in potato.

Taken together, the strong correlation between the amiRNA::*StTCP23* sequence complementarity and the severity of the developmental phenotype expressed by the VMR-derived amiRNA plants together with the lack of any visible phenotypes in the non-VMR amiRNA expressing plants, strongly indicates that amiRNA-directed RNA silencing of *StTCP23* is responsible for the phenotypes expressed by transformed potato plants. These results also suggest that the corresponding VMR-derived sRNAs in PSTVd-infected potato are responsible for the onset of disease symptom development by directing silencing of *StTCP23*. *St*TCP23 belongs to the TCP class of transcription factors, a group of transcription factor that play an important role in regulating plant growth and development, especially leaf development, cell proliferation in young internodes and specialized floral organs, and the development of branch and leaf shape [54–59]. While the function of TCP in potato has not been studied extensively, two recent reports have already demonstrated that *StTCP1* is involved in the control of meristem activation and *branched1a* encodes a TCP transcription factor that controls aerial and underground lateral shoot outgrowth, as well as tuber development in potato [60, 61].

TCP family genes share certain structural similarities with other proteins containing basic helix-loop-helix (bHLH) motifs that facilitate DNA binding and protein-protein interactions, and a small number of recent reports have shown that interaction of class 1 TCP transcription factors with DELLA proteins in the shoot apex of the inflorescence act to control plant height and reduced responsiveness to gibberellins [62, 63]. The abnormal phenotypes observed in the amiR45 and amiR46 transformed lines as well as in *StTCP23*-VIGS plants, are consistent with these findings, namely, down-regulation of *StTCP23* expression, and therefore, *StTCP23* activity in these plants resulted in stunting, leaf twisting and abnormal branching in the foliage as well as the formation of elongated, spindle-shaped tubers in their underground portions. GA promotes the ubiquitination and degradation of the growth-repressing DELLA proteins. Low levels of GA allow DELLA proteins to accumulate, and these proteins then bind to and inactivate a number of transcription factors having critical regulatory effects on plant development. Twenty percent of the proteins that have been demonstrated to interact with DELLA proteins belong to the TCP transcription factor family [64]. GA-induced DELLA degradation would therefore release these TCPs, thereby stimulating shoot elongation and seed germination respectively [65].

The formation and growth of the potato tuber is a complex process that is regulated by many hormones; in particular, the action of GA has been implicated in several different aspects of tuber formation. Onset of tuberization is strongly correlated with a drop in bioactive GA in subapical regions of the stolon, and early induction of the catabolic enzyme, *StGA2ox1*, plays a crucial role in this process [33]. However, an increased in bioactive gibberellins levels owing to specific expression of a biosynthetic GA3ox enzyme in the stolons resulted in only a very subtle effect on tuberization [66, 67]. Either the up-regulation of a gene involved in GA inactivation (*StGA2ox1*), or the down-regulation of a GA biosynthesis gene (*StGA3ox2*) would allow for a rapid reduction in GA content within the swelling stolon required for normal tuber formation [68].

Katsarou et al. have reported that PSTVd infection leads to down-regulation of *StGA7ox* expression in developing potato tubers [22]. Furthermore, the expression of one additional gene, *StGA20ox1*, involved in the synthesis of the GA precursor, GA20, has been shown to be under negative feedback control by GA via repression of the DELLA-GAF1 complex [69]. Differential expression of these GA metabolism genes in PSTVd infected plants provides new insight into the effects of PSTVd infection on GA metabolism and signaling during tuber development. For the first time, the effects of PSTVd infection were observed at every level in the signaling pathway in this study. How the effects of transcription factors such as *StTCP23* are propagated downstream to alter the expression and/or abundance of individual enzymes remains to be determined. Class I TCP transcription factors show a preference for interaction with *cis*-elements containing the sequences, TTGGGCC, GTGGG, GTGGGCCNNN and TGGGC [57, 62, 64, 70–72]. Considering that *St*TCP23 belongs to Class 1, we have examined the promoter region (arbitrarily defined as -2000 to -10 bp from the initial ATG start codon) of *StGA7ox* and five other GA-related genes assessed in our study for the presence of any of these elements. Each promoter contained at least two of these elements, but no single combination of elements could be correlated with either up- or down-regulated expression (S2 Table).

**Fig 8:**
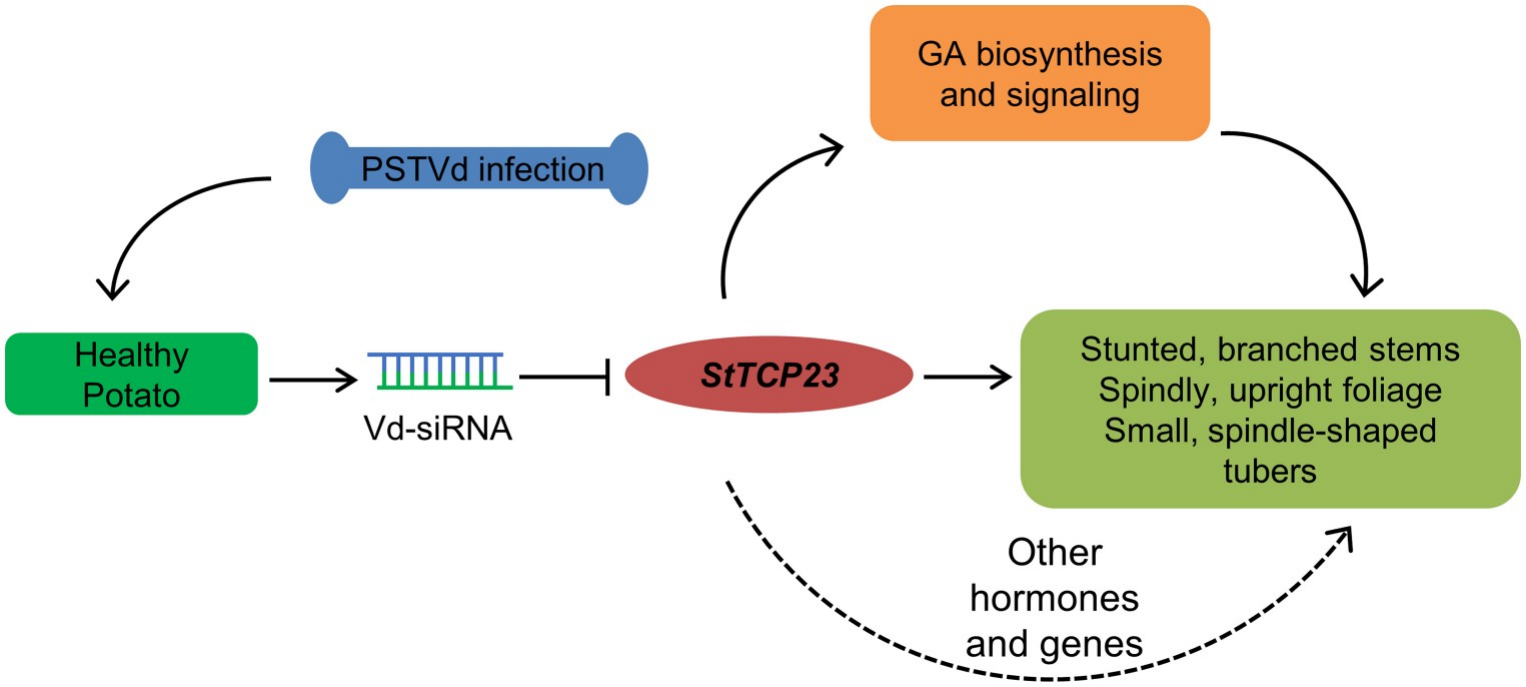
Effects of PSTVd-mediated silencing of *StTCP23* silencing on plant development. Solid lines, regulatory links observed in our experiments; dashed lines, possible regulatory links observed in other studies. Arrows indicate positive regulation; blunt ended bars indicate inhibition.

Our results provide the strongest evidence to date for a central role for RNA silencing in mediating disease induction upon PSTVd infection (and presumably by other pospiviroids). In potato tubers, reduced expression of the TCP transcription factor, *St*TCP23 directed by sRNAs derived from the VMR of PSTVd was shown to be accompanied by changes in the levels of transcripts encoding proteins involved in GA signaling as well as gibberellin biosynthesis/degradation, a decrease in GA concentration, and morphological changes very similar to those caused by paclobutrazole, a widely-used inhibitor of GA_3_ activity (Fig 8).

Additional studies are required to characterize (1) interactions between *StTCP23*, the DELLA protein GAI, and the gibberellin receptor GID1, and (2) the role played by *St*TCP23 in regulating the expression of key genes involved in GA biosynthesis and degradation. Of particular interest are possible differences in interactions occurring in the foliage versus those occurring in tubers. If sRNAs from the VMR do, in fact, play a key role in initiating the disease process associated with PSTVd infection, it may be possible to suppress their function and hence disease development using a sRNA sponge strategy in the future. While our studies provide strong evidence for the vd-sRNA-mediated disease mechanism in potato, the involvement of other sRNA-independent mechanisms in viroid diseases should also be considered given the many common features of disease symptoms by different viroids in plants.

## Methods

### PSTVd infection

A severe isolate of PSTVd (PSTVd-RG1; GeneBank accession number: U23058.1) was used to infect potato plants. Precisely full-length monomeric RG1 RNA transcripts were synthesized from Hind III-linearized plasmid PzTR8 using T7 RNA polymerase as described by Owens et al. [73]. Transcripts were quantified by UV spectrophotometry, and their integrity was confirmed by 1.5% agarose gel electrophoresis. Inocula were diluted to a final concentration of 100 ng/ul in 50 mM sodium phosphate buffer (pH 7.0). For mechanical inoculation, young leaves of potato plants at the 10-leaf stage were dusted with carborundum (600-mesh) before an aliquot (e.g., 10 μl) of inoculum was placed on the leaf and gently rubbed 10–20 times with a sterile glass bar. The inoculated plants were immediately rinsed with distilled water and incubated for two hours in a insect-free, air conditioned greenhouse controlled at a cool temperature before transfer to 25–30°C with high light intensity (fluorescent, 40 W×4, ca. 60 cm distance). Nine seedlings per transgenic line were used for each infection assay, and the assay was repeated tree times [13].

### Construction of PSTVd amiRNA vectors

Six PSTVd-specific amiRNA plant expression vectors were generated using the pBlueGreen amiRNA cassette that is based on the *A*. *thaliana* MIR159B precursor transcript as described by Eamens et al. [19]. Four of the six amiRNA vectors were designed to generate mature 21 nt amiRNA-sRNAs corresponding to VMR sequences that start at PSTVd-RG1 genome positions 45, 46, 47, or 50. The remaining two sequences start at positions 24 or 71 outside the VM region and acted as controls.

### Potato transformation and growth

Artificial miRNA vectors that contained the modified amiRNA precursor transcript (PRI-AMIRNA) in the desired 5ʹ-3ʹ orientation were introduced into *Agrobacterium tumefaciens* strain LB4404 via electroporation in the presence of the helper plasmid pSoup [74]. *Agrobacterium*-mediated transformation of potato (*Solanum tuberosum* L. cv. Atlantic) was conducted as previously described [75], using 10 mg/L phosphinothricin as the selective agent. Primary transformant lines were grown *in vitro* on MS (Murashige and Skoog) agar medium containing 3% sucrose and 0.6% agar, pH 5.7 ± 0.05. Young plants with 7–10 leaves were transferred to soil and grown in a 18–25°C greenhouse or net house under natural light. Standard PCR techniques and primers p35SP-F2 and p35SP-R1 (S1 Table) were used to identify transgene-positive segregants.

### Bioinformatic and phylogenetic analysis

The 21 nt sequences corresponding to the mature sRNAs processed from the six PSTVd-specific amiRNA plant expression vectors created for this study were used to interrogate publicly available *S*. *tuberosum* transcriptome datasets using their Basic Local Alignment Search Tool (BLAST) function. Datasets searched for potential PSTVd-specific amiRNA target sequences included: psRNATarget: A Plant Small RNA Target Analysis Server (http://plantgrn.noble.org/psRNATarget/), National Centre for Biotechnological Information (http://blast.ncbi.nlm.nih.gov/), Potato Genome Sequencing Consortium (http://solanaceae.plantbiology.msu.edu/pgsc_download.shtml), The Arabidopsis Information Resource (https://www.arabidopsis.org/portals/education/aboutarabidopsis.jsp). The free energy of potential duplexes between amiRNA sRNAs and their bioinformatically identified target gene mRNAs, represented as a delta G (ΔG) value, was determined using Pairfold (http://www.rnasoft.ca/cgi-bin/RNAsoft/PairFold/pairfold.pl). Guided by an updated classification of plant TCP transcription factors, full-length sequences of TCP genes from other species were downloaded from the corresponding databases to search for *St*TCP23 homologs. Multiple sequence alignments were carried out using ClustalW and plotted with Bioedit (http://www.mbio.ncsu.edu/bioedit/bioedit.html). Sequence identity was analyzed using MegAlign in DNASTAR (Lasergene7.1, USA). A neighbor-joining phylogenetic analysis (1000 bootstrap replicates) was performed using Molecular Evolutionary Genetics Analysis software (MEGA, version 7.0.14).

### RNA Extraction, RT-qPCR, and northern blot analysis

Total RNA was extracted from various potato tissues using RNAiso Plus TRIZOL reagent (TakaRa, Japan). Small RNAs were isolated using the Small RNA Isolation Kit (TaKaRa, Japan), and oligo (dT)-primed first strand cDNA synthesis was carried out using 1 ug total RNA and the PrimerScript RT reagent kit (Takara, Japan) according to the manufacturer’s instructions. Aliquots (10 ng) of the resulting cDNA were used for the subsequent RT-qPCR assays together with appropriate primer combinations. Gene-specific primers sets designed using the Primer 5 program are described in (S1 Table).

PCR reactions (20 μl) containing QuantiMix SYBR (Takara, Japan) were incubated as follows: 30 s at 95°C followed by 40 cycles of 5 s at 95°C, 31 s at 60°C. RT-qPCR was performed on a Quantstudio 7 Real Time PCR system (Applied Biosystems, USA), using SYBR Premix Ex Taq II (Takara, Japan) with gene-specific primers (S1 Table). To normalize RT-qPCR results, the potato actin-1 gene (PGSC0003DMT400071331) was used as an internal reference. CT values were obtained with the Real-Time PCR System StepOne version 2.1 software (Applied Biosystems). Relative fold expression changes were calculated by the comparative CT method: fold change was calculated as 2^-ΔΔCT^. Gene expression patterns were compared using heat maps generated with the software MultiExperiment Viewer.

Northern blot analyses were performed as previously described [19] using 3ʹ-^32^P end-labeled oligonucleotide probes. U6 and ribosomal RNAs (5S, 18S, and 28S) were used as internal controls. Probe sequences are listed in (S1 Table). As for small RNA hybridization, we ran all samples in the same gel, and blotted all RNA samples to the same hybridizing filter. We then cut the top part off the whole blot and hybridized it with the U6 RNA probe as loading control. For the bottom part containing the sRNAs, we separated the lanes, and hybridized each lane separately with the different vd-sRNA probes. The different hybridized strips were then assembled and presented together with the U6 hybridized image as shown in Fig 1C.

### 3ʹ RNA ligase-mediated rapid amplification of cDNA ends

RISC-mediated cleavage sites in TCP transcription factor *StTCP23* mRNA isolated from PSTVd-infected potato plants were identified by 3′ RLM-RACE. For 3ʹ RACE Exp.1, a 3ʹ adenylated, 3ʹ amine-containing oligodeoxynucleotide universal miRNA cloning linker (NEB, Inc, USA) was ligated to the free 3ʹ-hydroxyl end of cleaved RNAs. Specifically, 10 μg of total RNA was mixed with a universal miRNA cloning linker in the absence of ATP and incubated for 2 h at 37°C in the presence of T4 RNA ligase I. The ligation products were reverse transcribed using a linker- specific reverse primer, and the resulting cDNA products were subsequently amplified by nested PCR using *StTCP23* mRNA primers (S1 Table). The purified nested PCR products were cloned into the pGEM-T easy vector (Promega, USA) and commercially sequenced.

3ʹ RACE Exp.2 followed the protocol from Zuber et al. [25]. Twenty pmol of a 5-riboadenylated DNA oligonucleotide (3-Adaptor, S1 Table) were ligated to 10 μg of total RNA using 20 U of T4 RNA Ligase 1 (NEB, Inc, USA) in a final volume of 100 μl for 1 h at 37°C and 1X T4 of RNA Ligase Reaction Buffer. The adapter-ligated mRNAs were then recovered by phenol/chloroform extraction followed by ethanol precipitation. cDNA synthesis was performed in two 20 μl-reactions for each sample. Each 20 μl reaction contained 2 μg of purified ligated RNA, 50 pmol of the RT oligonucleotide (S1 Table). Reactions were incubated at 50°C for 10 min, and then at 80°C for 10 min to inactivate the reverse transcriptase. 3ʹ RACE amplifications were performed using the gene-specific primers, and the GeneRacer 3′ primers (S1 Table) after the vd-sRNA-cleaved target mRNA libraries were prepared. The amplification products were gel purified and cloned. Six independent clones were sequenced, and the resulting sequences clones were aligned to the predicted *StTCP23* mRNA sequences for detection of splicing sites.

### Construction of TRV VIGS vectors and agroinfiltration

The VIGS vectors pTRV1 (RNA1) and pTRV2-LIC 2.0 beta (pYY13) used for VIGS of *StTCP23* were kindly provided by Dr Yule Liu (Tsinghua University, Beijing, China). The pTRV2-LIC 2.0 beta vector containing TRV RNA2 was used in silencing experiments to express a partial sequence of *StTCP23* gene amplified by specific primers (S1 Table). The resulting PCR products were ligated into pTRV after cleavage with the appropriate enzymes as described previously [76–79]. The pssRNAit server (http://plantgrn.no-ble.org/pssRNAit/) together with the *Solanum tuberosum* potato unigene [DFCI Gene Index (STGI), "version 13 released on 2010_04_16] and potato transcript [Group Phureja DM1-3 516R44 (CIP801092) Genome 3.4 transcripts] databases were used to detect potential siRNA off-targeting. Sequences lacking problematic regions were chosen for VIGS experiments. A *PDS* gene construct in pTRV: *PDS* was used for control treatment.

TRV infection by *Agrobacterium tumefaciens* strain GV3101 infiltration of potato variety cv. Atlantic was performed as previously described [80]. To confirm systemic infection by TRV, viral RNAs in newly emerged leaves were amplified by RT-qPCR with primers designed from the TRV capsid protein (CP)-coding gene. The *Actin-1* gene of potato (PGSC0003DMT400071331) served as internal reference.

### GFP transient expression vector and agro-infiltration

To confirm the targeting of VM-siRNA46 to *StTCP23* transcripts, a green fluorescent protein (GFP) tagged target construct was prepared by ligating the 21 nt predicted target sequence (*StTCP23*-3ʹ UTR) into the 3ʹ-untranslated region (3ʹ UTR) of the GFP gene under the control of the 35s promoter in pCAMBIA1300-35S-GFP vector as previously described [81, 82]. The transient miRNA expression construct was created in pGreen II 62-SK as described [83]. The GFP construct was co-agro-infiltrated into *Nicotiana benthamiana* leaves together with either the targeting amiRNA46 construct or an empty vector. At 5dpi, agro-infiltrated leaves were observed under UV illumination, and GFP fluorescence was recorded.

### Quantification of endogenous GA and chemical treatments

Quantitative analyses of endogenous GAs were carried out on samples of tuber tissue collected from transgenic clones (amiR45, amiR46, amiR71) as well as healthy and PSTVd-infected non-transgenic cv. Atlantic plants. Tubers (three independent replicates) were harvested from 3-month-old plants and immediately frozen in liquid nitrogen before being ground into a fine powder. A portion (0.1gm fresh weight) of each sample was extracted with 80% methyl alcohol, and GAs were quantified by High Performance Liquid Chromatography (HPLC, Rigol L3000) on a Kromasil C18 reversed-phase chromatographic column (250 mm x 4.6 mm, 5 μm) using selected ion monitoring.

Stock solutions of GA_3_ (500 uM, Sigma) and paclobutrazol (500 mg/L, Sigma) were prepared in 100% ethanol containing 0.02% (v/v) Tween 20. Month-old young plants growing in soil were sprayed at two weeks intervals with either GA_3_ (5 uM) or paclobutrazol (50 mg/L), a GA_3_ biosynthesis inhibitor. Control plants were sprayed with distilled water containing 0.02% (v/v) Tween 20. Tubers were harvested and evaluated 4 months after planting.

### Measurement of pollen viability

Pollen viability was measured using pollen grains collected from three flowers per plant. Viability rates (%) were calculated using automatic and manual pollen grain counting for six amiRNA transgenic and one control line.

### Statistical analysis

All data were analyzed by ANOVA (analysis of variance) and the Student’s t-test, where n = 18. Error bars indicate ± SE (standard error) as determined by the Origin8 program. Statistical differences were considered significant at p < 0.05 (*), p < 0.01 (**).

## Supporting information

S1 Fig Differential RT-qPCR amplification of *StTCP23* mRNA around the sRNA-binding site is consistent with vd-sRNA-mediated cleavage. (A) Schematic diagram of RT-qPCR primer design. (B) RT-qPCR amplification result. The region downstream of the predicted vd-sRNA-binding site in *StTCP23* mRNA (PCR3) shows higher ratios of RT-qPCR amplification than the region upstream of (PCR2) or spanning (PCR1) the binding site. The value of PCR1 was set as 1. Left panel: oligo-dT primer was used for reverse transcription. Right panel: random hexamer primers was used for reverse transcription.
(TIF)

S2 Fig Sequence complementarity between *StTCP23* mRNA and the upper portion of PSTVd V(irulence) M(odulating) region. Green: G-U wobble base pair; red: mismatch. Lethal PSTVd strains RG1 and KF440-2 differ from intermediate and mild strains at positions 46 (C) and 47 (A) where these positions are occupied by G and C, respectively. Lethal strain AS1 differs at position 47 (U).
(TIF)

S3 Fig Developmental effects of PSTVd amiRNA expression in transgenic potato. (A) Branched phenotypes were observed in amiR46 transgenic lines. (B) The above-ground portions of these plants matured early, and tubers developed more rapidly than for non-transgenic controls, Total lifespan from emergence of the first shoot to senescence and death of the mature plant was only two months. (C) Abnormal anther development in several lines expressing PSTVd amiRNAs. (D) Pollen viability measured using pollen grains collected from three flowers per plant. Viability rates (%) were calculated using both automatic and manual pollen grain counting for six amiRNA transgenic lines and wild type control.
(TIF)

S4 Fig Heat map representation of *StTCP23* expression in different tissues. The three heat maps are derived from either FPKM data from the PGSC database (A = DM, B = RH) or RT-qPCR analysis of cv. Atlantic (C). All RT-qPCR experiments were performed using three biological replicates and with three technical replicates. The relative expression levels for each gene were calculated using 2^−ΔΔCT^ method in comparison with the control gene. Relative expression values were transformed to log2 (value +1), and the number was represented by the color bar, red as higher expression levels and blue as lower expression levels.
(TIF)

S5 Fig Heat map representation comparing the expression levels in leaf tissue of different amiRNAs. From left to right, three-time course (1, 2, 3 month) of PSTVd-infected non-transgenic plants and amiRNAs transgenic Line (amiR24-2, amiR45-14, amiR46-4, amiR47-14, amiR50-12, amiR71-6 was used for relative amiRNA expression respectively) followed by uninfected non-transgenic cv. Atlantic plants. All RT-qPCR experiments were performed using three biological replicates and with three technical replicates. Expression levels in all samples were compared with that in one- month infected cv. Atlantic plants (2^-ΔΔCT^ = 1) by RT-qPCR analysis, and relative expression levels were transformed to log2 (value +1). The color scale representing the relative signal values is shown at the upper right (blue; low expression, yellow; medium expression, and red; high expression). Red triangle on the right side of the figure indicate accession numbers for *StTCP23*.
(TIF)

S6 Fig Heat map representation comparing the expression levels in leaf tissue of potential PSTVd VM sRNA target genes. From left to right, three-time course (1, 2, 3 month) of uninfected non-transgenic cv. Atlantic plants and amiRNAs transgenic Line (amiR24-2, amiR45-14, amiR46-4, amiR47-14, amiR50-12, amiR71-6) followed by PSTVd-infected non-transgenic plants. All RT-qPCR experiments were performed using three biological replicates and with three technical replicates. Expression levels in all samples were compared with that in one- month uninfected cv. Atlantic plants (2^-ΔΔCT^ = 1) by RT-qPCR analysis, and relative expression levels were transformed to log2 (value +1). The color scale representing the relative signal values is shown at the upper right (blue; low expression, yellow; medium expression, and red; high expression). Red triangle on the right side of the figure indicate accession numbers for *StTCP23*.
(TIF)

S7 Fig Phylogenetic analysis of TCP family members from *Solanum tuberosum*, *Arabidopsis thaliana*. (A) An unrooted neighbor-joining phylogenetic tree was constructed from an unadjusted ClustalW alignment of the full-length amino acid sequences of 23 potato and 24 Arabidopsis TCP proteins downloaded from PGSC (http://solanaceae.plantbiology.msu.edu/) and PlantTFDB (http://planttfdb.cbi.pku.edu.cn/) respectively, using MEGA 6.0 and 1000 bootstrap replications. Three TCP proteins from *A*. *majus* and one from *Z*. *mays* were included as controls. The three resulting clades (CYC, PCF, CIN) are shaded in different colors. (B) Comparison of tuber number and (C) average tuber weight for treatment with ethanol, PBZ, or GA_3_.
(TIF)

S1 Table Oligonucleotides used in this study. (DOCX)

S2 Table Putative TCP binding sites in promoters of genes involved in GA metabolism. (DOCX)

S1 File Predicted target genes for the different PSTVd sRNAs corresponding to the vd-sRNAs and their expression patterns in PSTVd-infected and amiRNA potato plants. (XLSX)
