# Supplementary figures and images for: Silencing of transcription factor encoding gene StTCP23 by small RNAs derived from the virulence modulating region of potato spindle tuber viroid is associated with symptom development in potato
Source: PLoS Pathog. 2019 Dec 2;15(12):e1008110. doi: 10.1371/journal.ppat.1008110 (PMC6907872; doi:10.1371/journal.ppat.1008110)

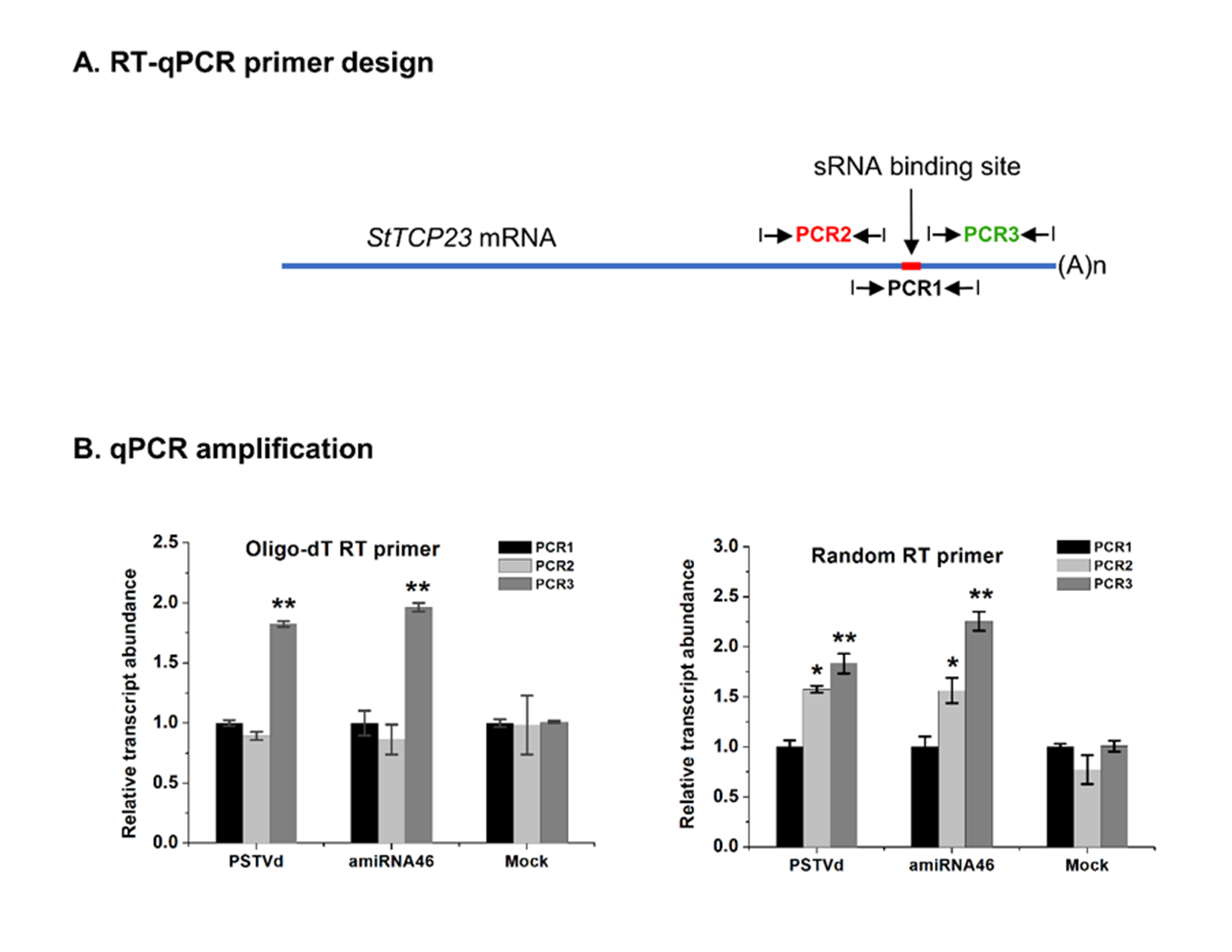

Supplement: S1 Fig — (A) Schematic diagram of RT-qPCR primer design. (B) RT-qPCR amplification result. The region downstream of the predicted vd-sRNA-binding site in StTCP23 mRNA (PCR3) shows higher ratios of RT-qPCR amplification than the region upstream of (PCR2) or spanning (PCR1) the binding site. The value of PCR1 was set as 1. Left panel: oligo-dT primer was used for reverse transcription. Right panel: random hexamer primers was used for reverse transcription. (TIF) [file ppat.1008110.s001.tif]

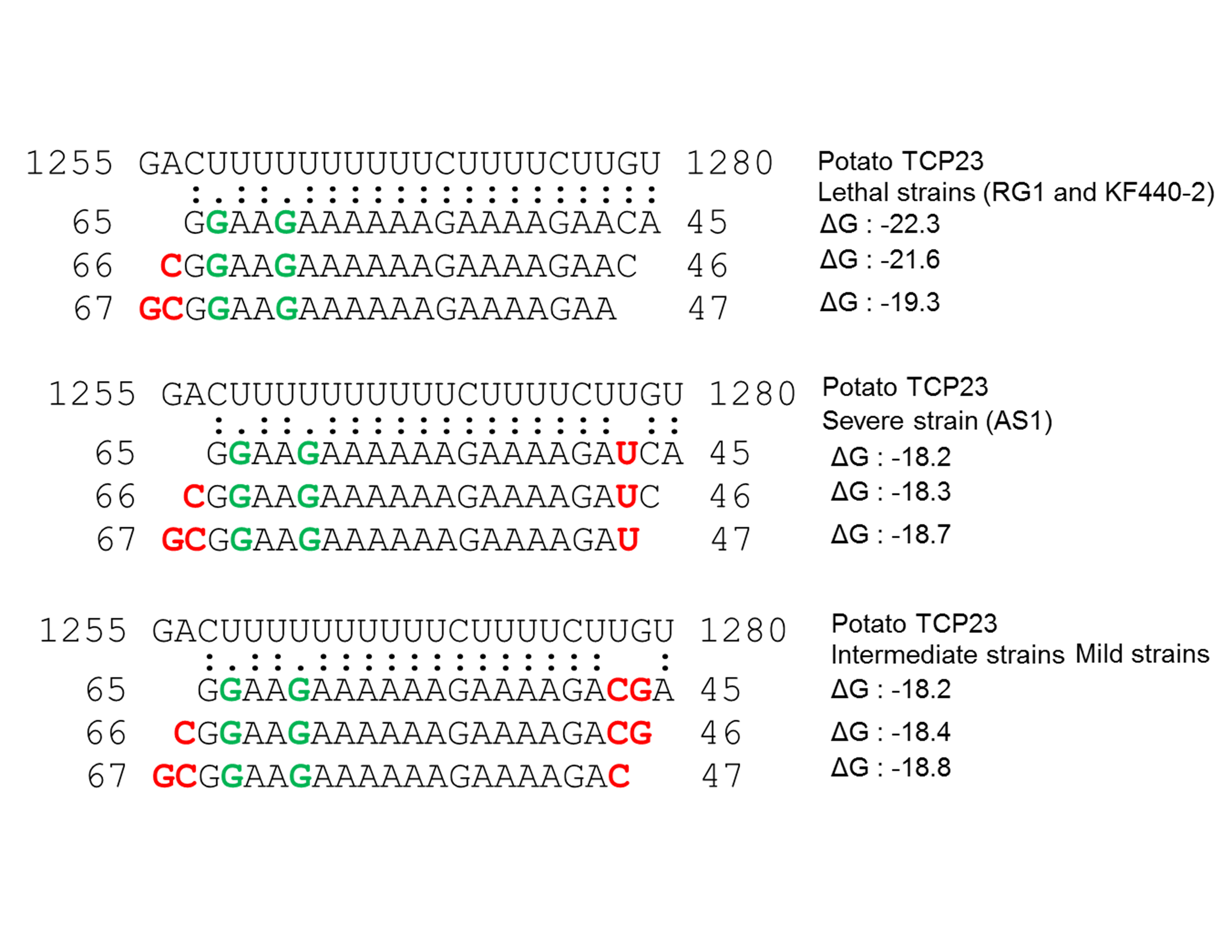

Supplement: S2 Fig — Green: G-U wobble base pair; red: mismatch. Lethal PSTVd strains RG1 and KF440-2 differ from intermediate and mild strains at positions 46 (C) and 47 (A) where these positions are occupied by G and C, respectively. Lethal strain AS1 differs at position 47 (U). (TIF) [file ppat.1008110.s002.tif]

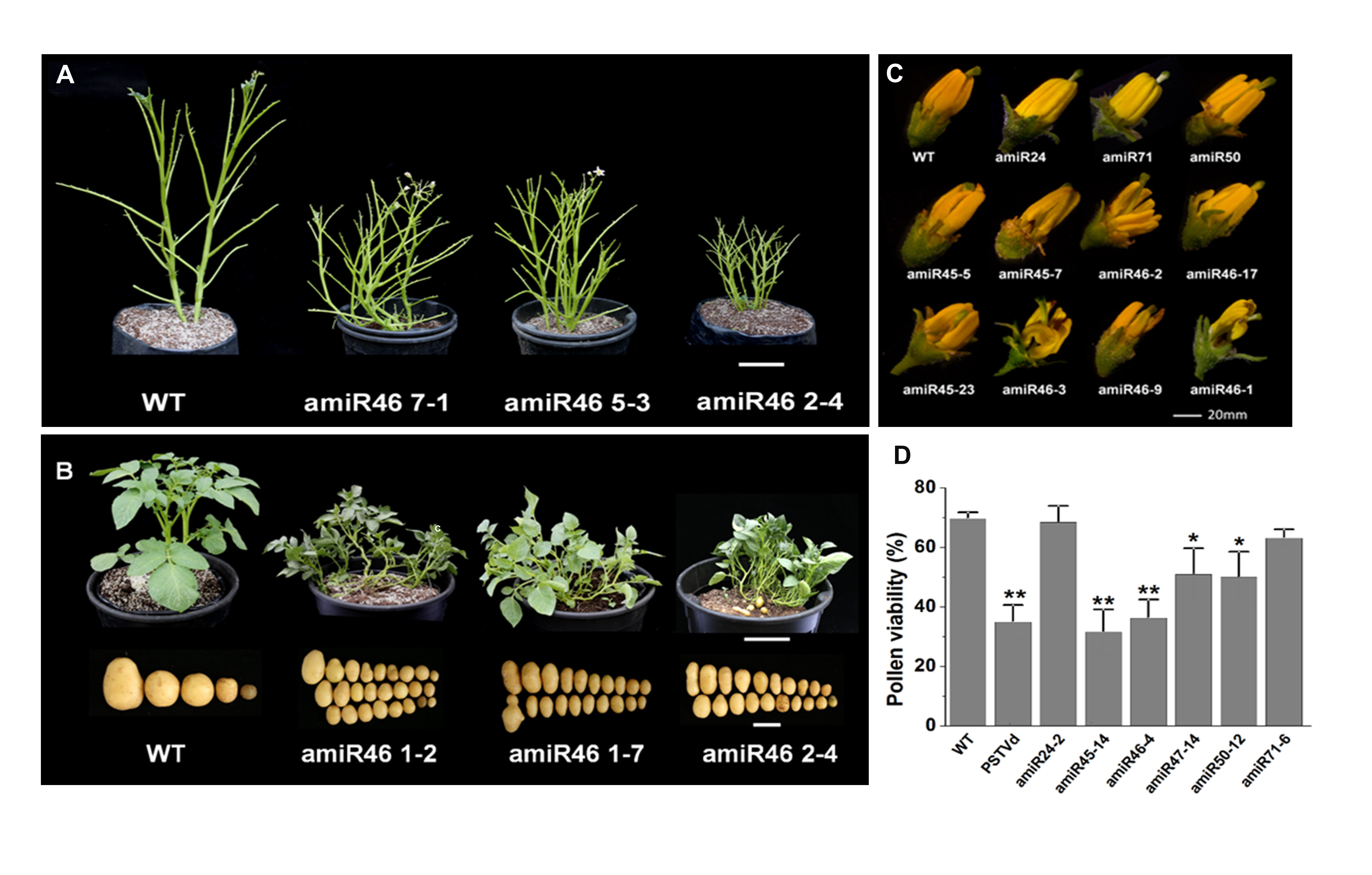

Supplement: S3 Fig — (A) Branched phenotypes were observed in amiR46 transgenic lines. (B) The above-ground portions of these plants matured early, and tubers developed more rapidly than for non-transgenic controls, Total lifespan from emergence of the first shoot to senescence and death of the mature plant was only two months. (C) Abnormal anther development in several lines expressing PSTVd amiRNAs. (D) Pollen viability measured using pollen grains collected from three flowers per plant. Viability rates (%) were calculated using both automatic and manual pollen grain counting for six amiRNA transgenic lines and wild type control. (TIF) [file ppat.1008110.s003.tif]

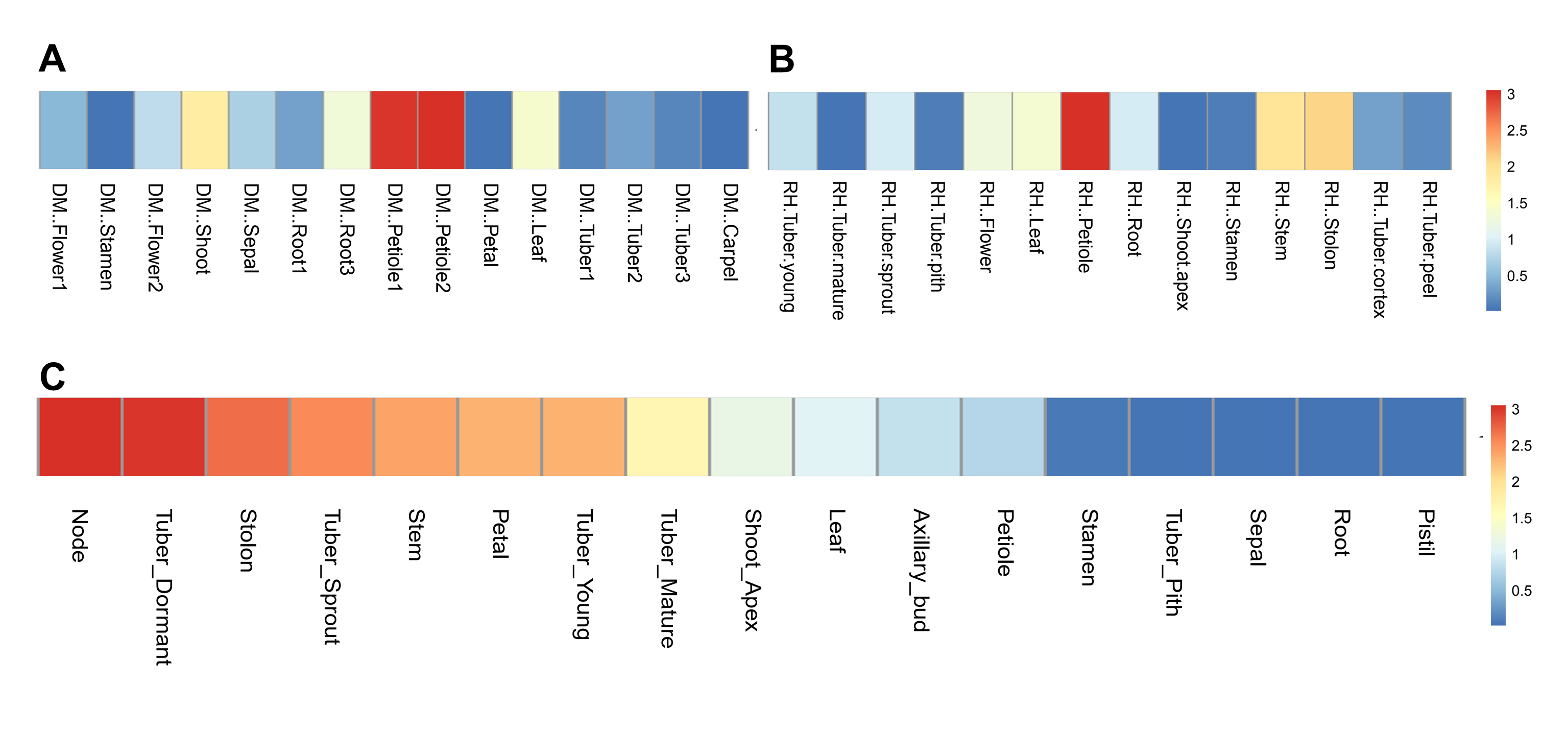

Supplement: S4 Fig — The three heat maps are derived from either FPKM data from the PGSC database (A = DM, B = RH) or RT-qPCR analysis of cv. Atlantic (C). All RT-qPCR experiments were performed using three biological replicates and with three technical replicates. The relative expression levels for each gene were calculated using 2−ΔΔCT method in comparison with the control gene. Relative expression values were transformed to log2 (value +1), and the number was represented by the color bar, red as higher expression levels and blue as lower expression levels. (TIF) [file ppat.1008110.s004.tif]

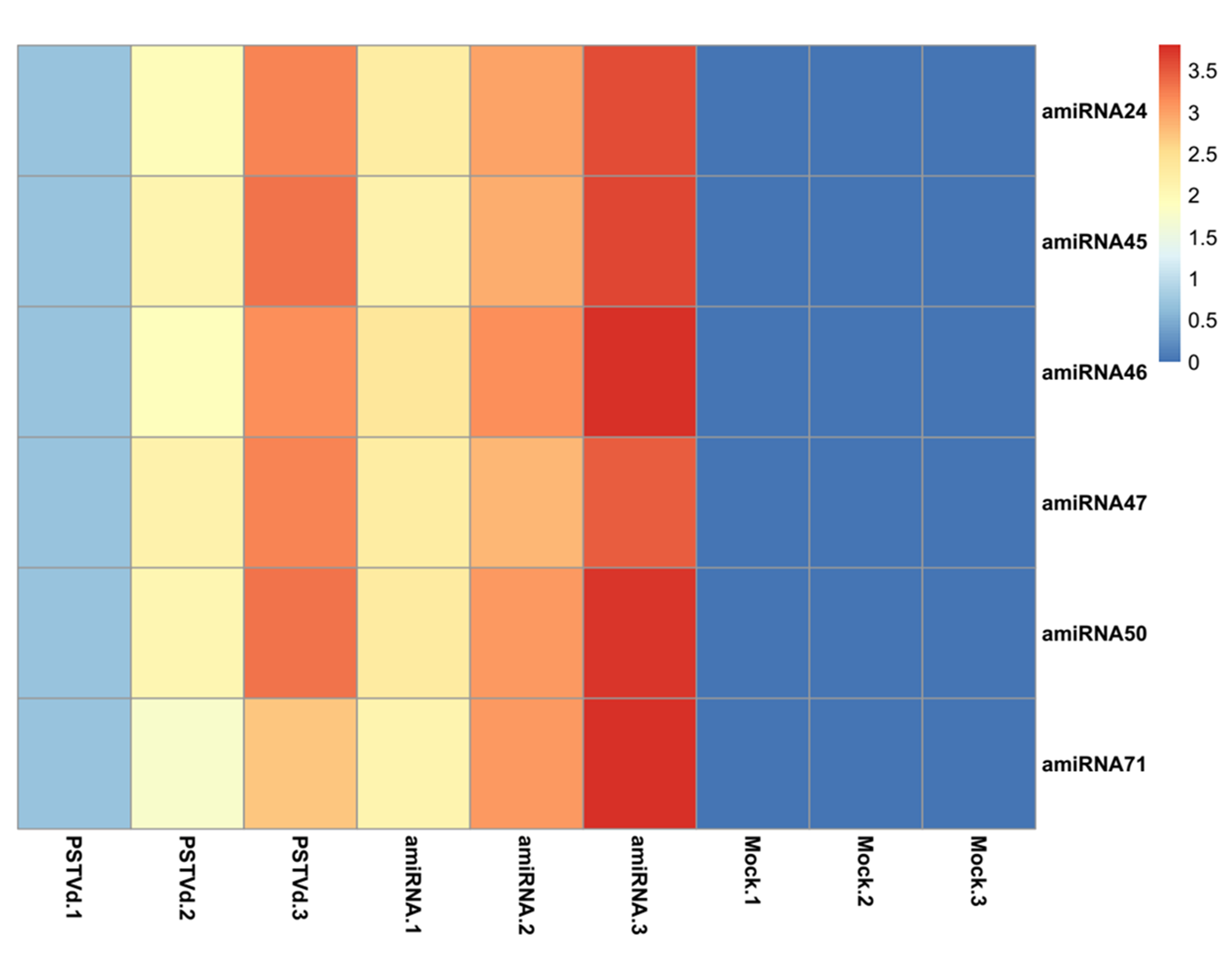

Supplement: S5 Fig — From left to right, three-time course (1, 2, 3 month) of PSTVd-infected non-transgenic plants and amiRNAs transgenic Line (amiR24-2, amiR45-14, amiR46-4, amiR47-14, amiR50-12, amiR71-6 was used for relative amiRNA expression respectively) followed by uninfected non-transgenic cv. Atlantic plants. All RT-qPCR experiments were performed using three biological replicates and with three technical replicates. Expression levels in all samples were compared with that in one- month infected cv. Atlantic plants (2-ΔΔCT = 1) by RT-qPCR analysis, and relative expression levels were transformed to log2 (value +1). The color scale representing the relative signal values is shown at the upper right (blue; low expression, yellow; medium expression, and red; high expression). Red triangle on the right side of the figure indicate accession numbers for StTCP23. (TIF) [file ppat.1008110.s005.tif]

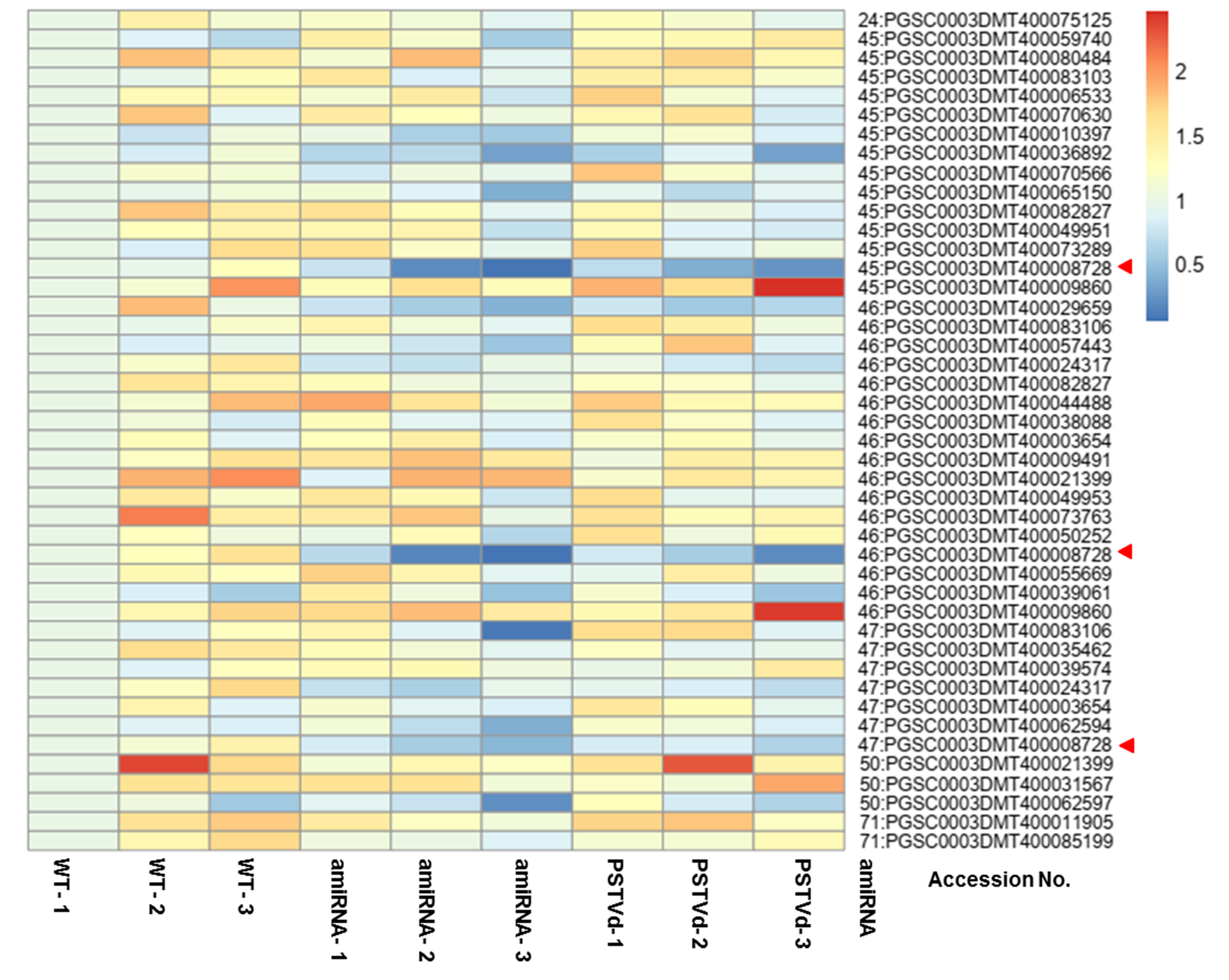

Supplement: S6 Fig — From left to right, three-time course (1, 2, 3 month) of uninfected non-transgenic cv. Atlantic plants and amiRNAs transgenic Line (amiR24-2, amiR45-14, amiR46-4, amiR47-14, amiR50-12, amiR71-6) followed by PSTVd-infected non-transgenic plants. All RT-qPCR experiments were performed using three biological replicates and with three technical replicates. Expression levels in all samples were compared with that in one- month uninfected cv. Atlantic plants (2-ΔΔCT = 1) by RT-qPCR analysis, and relative expression levels were transformed to log2 (value +1). The color scale representing the relative signal values is shown at the upper right (blue; low expression, yellow; medium expression, and red; high expression). Red triangle on the right side of the figure indicate accession numbers for StTCP23. (TIF) [file ppat.1008110.s006.tif]

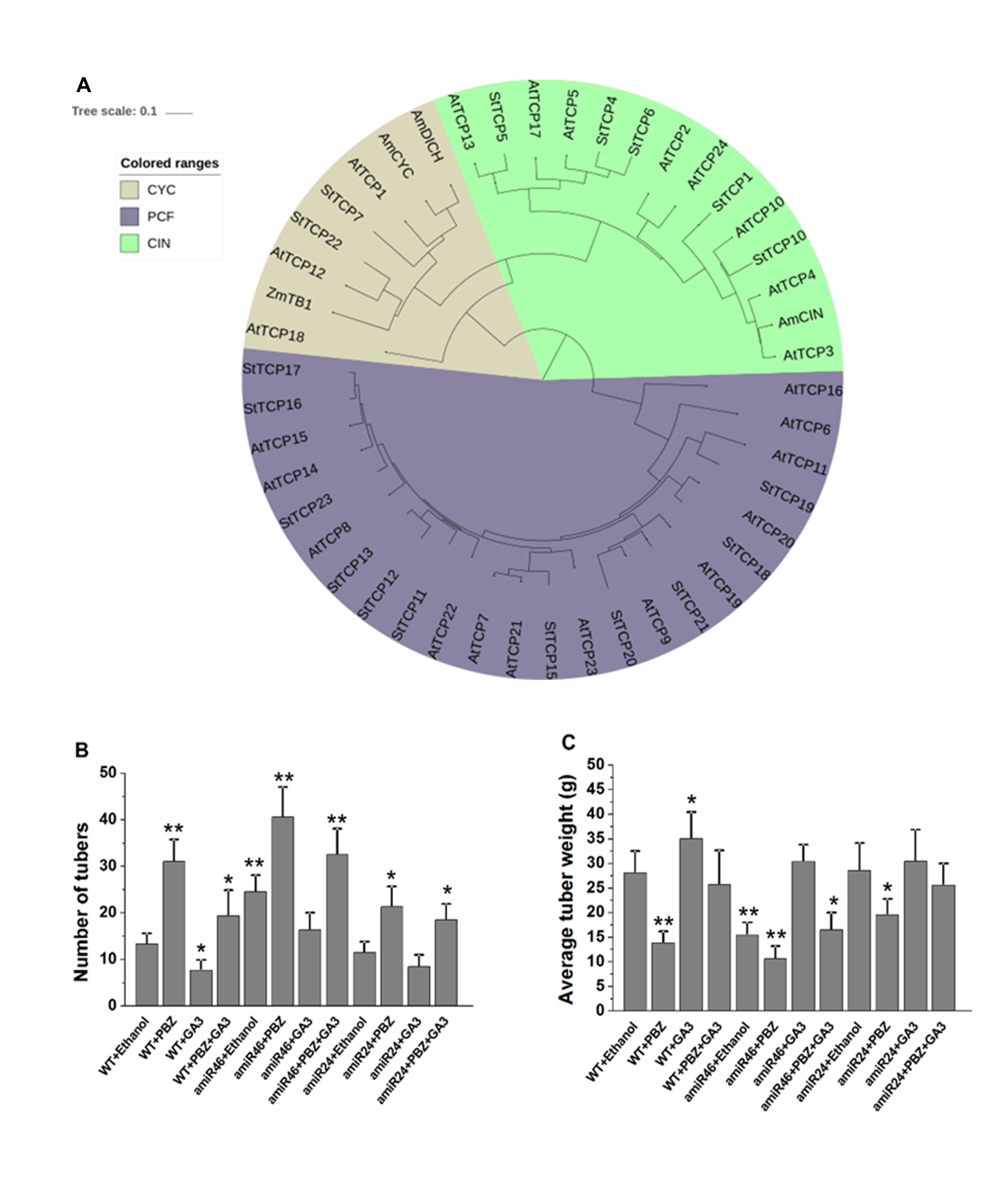

Supplement: S7 Fig — (A) An unrooted neighbor-joining phylogenetic tree was constructed from an unadjusted ClustalW alignment of the full-length amino acid sequences of 23 potato and 24 Arabidopsis TCP proteins downloaded from PGSC (http://solanaceae.plantbiology.msu.edu/) and PlantTFDB (http://planttfdb.cbi.pku.edu.cn/) respectively, using MEGA 6.0 and 1000 bootstrap replications. Three TCP proteins from A. majus and one from Z. mays were included as controls. The three resulting clades (CYC, PCF, CIN) are shaded in different colors. (B) Comparison of tuber number and (C) average tuber weight for treatment with ethanol, PBZ, or GA3. (TIF) [file ppat.1008110.s007.tif]
