# Supplementary material for: Silencing of transcription factor encoding gene StTCP23 by small RNAs derived from the virulence modulating region of potato spindle tuber viroid is associated with symptom development in potato
Source: PLoS Pathog. 2019 Dec 2;15(12):e1008110. doi: 10.1371/journal.ppat.1008110 (PMC6907872; doi:10.1371/journal.ppat.1008110)
Supplement: S1 Table — (DOCX) [file ppat.1008110.s008.docx]

**S1 Table. Oligonucleotides used in this study.**

| **Experiment** | **Target gene** | **Forward primer (5ʹ to 3ʹ)** | **Reverse primer (5ʹ to 3ʹ)** |
| --- | --- | --- | --- |
| Primers for quantitative RT-PCR | PSTVd-RG1 | CACACCTGACCTCCTGACAAG | GCACTCCCCACCGTCCTTT |
|  | DELLA | GGAATAGGCCCTCCACAACC | TGAAGCCGAAGACGAAGACC |
|  | GIDI | FTACATTTTGCCGCCGCCT | CCCATTTTAGAGCAGCCCATC |
|  | StGA20ox1 | CGGCCCAACAAGCATCTAAG | AAGCCATGACTCCGACACG |
|  | StGA20ox3 | GCAATGCCATGAGCACCC | GGCTCAATCCCAAAAGTTCCA |
|  | StGA3ox2 | AGCTCATGTGGTCCGAAGGA | CGGACAAGCCGGGTAAGAAT |
|  | StGA2ox1 | AGGCACAGAGTGATCGCAGA | TGGTGGCCCTCCAAAGTAAA |
|  | StGA7ox | TTTGAGCACTTGTGGTTTCG | TGCTTGAAATAAGGTGTAATGCT |
|  | Actin-1 | GATGGCAGACGGAGAGGA | GAGGACAGGATGCTCCTC |
|  | TRV-CP | TCATTTGACAAGTCGGGC | TGTGTTTGGATTCGCAGG |
|  | GFP: StTCP23 | CTGACGTAAGGGATGACGCA | TGGTGTGTGCGCAATGAAAC |
|  | p35S | GAGGATCTAACAGAACTCGC | GGAACGTCTTCTTTTTCCACG |
|  | StTCP23 | CAGCAACATGTGCAGCTAGG | TATACGAGTTCATCGCCGCC |
|  | StTCP23-PCR1 | GCGAGTCATCAAATTGGGCA | GTATTTAACTTCCCCATACAAGA |
|  | StTCP23-PCR2 | TGGACGAATTAAGCGGAGGTC | GCCCAATTTGATGACTCGCTG |
|  | StTCP23-PCR3 | GGGGAAGTTAAATACAATAGT | CAAGAAATTTATTTTGCCCTA |
|  | **amiRNAs** | **Forward primer (5ʹ to 3ʹ)** | **Reverse primer (5ʹ to 3ʹ)** |
|  | amiR24 | CGCGTGGTTCACACCTGAC | AGTGCAGGGTCCGAGGTATT |
|  | amiR45 | GCGCGACAAGAAAAGAAAAA | AGTGCAGGGTCCGAGGTATT |
|  | amiR46 | GCGCGCAAGAAAAGAAAAAA | AGTGCAGGGTCCGAGGTATT |
|  | amiR47 | CGCGCGAAGAAAAGAAAAAAG | AGTGCAGGGTCCGAGGTATT |
|  | amiR50 | CGCGCGAAAAGAAAAAAGAAG | AGTGCAGGGTCCGAGGTATT |
|  | amiR71 | GCGGAGGAGCGCTTCC | AGTGCAGGGTCCGAGGTATT |
|  | U6 | AGGGGCCATGCTAATCTTCTC | AGTGCAGGGTCCGAGGTATT |
| **3ʹ RLM RACE-Exp.1** | 3ʹ-Adapter | rAppCTGTAGGCACCATCAAT-NH2 | |
|  | StTCP23^a^-RACE F | GGCATATTCCAGCAACAACG (Primer binding site: 887-906) | |
|  | StTCP23-RACE nested F | AGACAAAGTGGTGGTCATGCT (Primer binding site: 1132-1152) | |
|  | 3ʹ RACE R^b^ | AATGATACGGAGACCACCGACAGATTGATGGTGCCTAC | |
|  | 3ʹ RACE nested R | ACCGACAGATTGATGGTGCC | |
| **3ʹ RLM RACE-Exp.2** | 3ʹ-Adapter | /5rApp/CTGACNNNNNNNNNNNNNNNTGGAATTCTCGGGTGCCAAGGC/3ddC/ | |
|  | 3ʹ-RT | GCCTTGGCACCCGAGAA | |
|  | StTCP23-RACE F | GGCATATTCCAGCAACAACG (Primer binding site: 887-906) | |
|  | StTCP23-RACE nested F | AATGATACGGCGACCACCGAGATCTACACGTTCAGAGTTCTACAGTCCGACGAT CAGAAGGGCAATGGGGGATG  (Primer binding site: 1088-1107) | |
|  | 3ʹ RACE R | CCTTGGCACCCGAGAATTCCA | |
|  | Illumina TruSeq RPI primers for RISC-fragment analysis | CAAGCAGAAGACGGCATACGAGATTTACGTGTGACTGGAGTTCCTTGGCACCCGAGAATTCCA | |
| **VIGS** | TRV: StTCP23 | CgagctcCGAATTAAGCGGAGGTCAGAAGA | CggatccTTGTCTATACGAGTTCATCGCC |
|  | Sequences of fragments inserted into TRV vector for VIGS | CGAATTAAGC GGAGGTCAGAAGATCAAGAC TTACTACAAA ATAATTACCCTATGAGTAATATGTTACAATCTAGTACTTATGGTTCAATT CCAGCGAGTCATCAAATTGG GCATATTCCA GCAACAACGT TATATATGATGACAAATAATAATAATAATT CTAGTCATCATGACCCTAATAATTGTTCTATGTGGGGATCTTCTAGTAATAATAATAATATTGAGAATTCAAATATTAGAGGAGGGTTAT TGAATTTTATGAATTTTCAT CAACCAATTTTGGGGACACG TGGTGGCGGTGGGACGGCGGCAGAAGGGCAATGGGGGATG TTGACGGCGGCGATGAACTCGTATAGACAA | |
| **Probes used for small RNA northern blots** | amiR24 | CAGGAGGTCAGGTGTGAACCA | |
|  | amiR45 | CCTTCTTTTTTCTTTTCTTGT | |
|  | amiR46 | GCCTTCTTTTTTCTTTTCTTG | |
|  | amiR47 | CGCCTTCTTTTTTCTTTTCTT | |
|  | amiR50 | TGCCGCCTTCTTTTTTCTTTT | |
|  | amiR71 | GATCCCTGAAGCGCTCTCCG | |
|  | U6 | AGGGGCCATGCTAATCTTCTC | |

a *Solanum tuberosum* PGSC Acc. PGSC0003DMT400008728.

b. RT and reverse primer for first round of PCR.
