# Supplementary material for: Silencing of transcription factor encoding gene StTCP23 by small RNAs derived from the virulence modulating region of potato spindle tuber viroid is associated with symptom development in potato
Source: PLoS Pathog. 2019 Dec 2;15(12):e1008110. doi: 10.1371/journal.ppat.1008110 (PMC6907872; doi:10.1371/journal.ppat.1008110)
Supplement: S2 Table — (DOCX) [file ppat.1008110.s009.docx]

**S2 Table**. **Putative TCP binding sites in promoters of genes involved in GA metabolism.**

| **Transcript ID** | **Gene Name** | **TTGGGCC** | **GTGGG** | **GTGGGCCNNN** | **TGGGC** |
| --- | --- | --- | --- | --- | --- |
| PGSC0003DMT400014555 | *StGA3ox2* | N | Y | N | Y |
| PGSC0003DMT400086432 | *StGA20ox1* | Y | Y | N | Y |
| PGSC0003DMT400029280 | *StGA2ox1* | N | Y | N | N |
| PGSC0003DMT400001607 | *StGA7ox* | Y | Y | N | Y |
| PGSC0003DMT400049448 | *DELLA-GAI* | N | Y | N | Y |
| PGSC0003DMT400022327 | *GID1* | N | Y | N | Y |

Notes: N, no putative TCP binding site in the promoter region of this gene; Y, one or more putative TCP binding sites in the promoter region of this gene. Promoter regions arbitrarily defined as sequences located -2000 to -10 bp relative to the ATG start codon.
